# Pyrometallurgical valorization of waelz, fayalite, and linz-donawitz slag mixtures

**DOI:** 10.1038/s41598-026-44763-3

**Published:** 2026-03-19

**Authors:** Junnile L. Romero, Volker Recksiek, Rafaela Debastiani, Md Naziat Hossain, Ludwig Blenau, Alexandros Charitos, Ari O. Vaisanen, Ajay B. Patil

**Affiliations:** 1 Helmholtz Zentrum Dresden Rossendorf – Helmholtz Institute Freiberg for Resource Technology (HZDR-HIF), Chemnitzer Strasse 40, Freiberg, Germany; 2 Faculty of Science and Mathematics, Department of Chemistry, The University of Jyväskylä, P.O. Box 35, Jyvaskyla, FI-40014 Finland; 3 Institute of Nonferrous Metallurgy and Purest Materials (INEMET), Technische Universität Bergakademie Freiberg (TUBAF), Leipziger Straße 34, Freiberg, Germany

**Keywords:** Circular-economy, Waelz slag, Fayalite Slag, LD Slag, Smelting, Pyrometallurgy, Engineering, Environmental sciences, Materials science

## Abstract

Waelz, Linz-Donawitz, and Fayalite slags are one of the largest industrial byproducts. They are presently under-utilized, and ending up in landfill. This work demonstrates that mixtures of these slags can be directly valorized, given that the combined composition has basicity (CaO/SiO₂ = 0.9–1.0) without the addition of fresh fluxes. Furthermore, the current work contributes to a deeper understanding of slag behavior by linking basicity and viscosity to interfacial tension and by constructing an isoviscosity-ternary phase diagram of CaO-SiO_2_-Al_2_O_3_. The current methodology gives a more comprehensive approach for designing self-fluxing slag mixtures that is feasible for smelting. X-ray computed tomography (XCT) measurements of the smelted products show the internal micro-structure, which provided insights into the correlations between viscosity, interfacial-tension, and the performance of each mixture. The metal product qualifies as vanadium-bearing pig iron based on the elemental analysis while the slag met the criteria for non-hazardous, construction-grade material according to the specifications of the United States, United Kingdom and Germany. Additionally, the roasted slag contains phases suitable for ceramic and porcelain applications, broadening its commercial application. The study illustrates a practical implementation of circular-economy principles for the metal industry and for metallurgical waste management by demonstrating electrified pyrometallurgy based on the induction furnace.

**Supplementary Information:**

The online version contains supplementary material available at 10.1038/s41598-026-44763-3.

## Introduction

Zinc, copper, and steel demand continues to rise, and with it, the generation of industrial by-products, such as slags^1–3^. Waelz, Linz-Donawitz, and Fayalite slags annual generation is about 2 million, 100 million, and 44 million tons, respectively^4–8^ making them one of the largest industrial by-products. Slags are partially utilized as construction material; however, the presence of hazardous components and tightening regulations prevents these slags for different applications^9–11^. These slags possess environmental threat due to heavy metal content, such as lead (Pb), manganese (Mn), zinc (Zn), and vanadium (V), which may potentially be released to the environment through weathering or harsh conditions^4,12,13^. Meanwhile, these metals are necessary for industrial progress and can be recovered from slags, highlighting their potential as a secondary resource. With only partial utilization of these slags and large volumes being landfilled, leads to wasting resources and causes long-term environmental issues.

Reductive smelting is one of the main techniques in producing metals, and fluxing plays an essential role in it. Because it decides the slag’s chemical and physical characteristics at defined process temperatures^14^. Commonly, fluxes can either be basic (e.g., calcium oxide-rich) or acidic (e.g., silica sand-rich), and the choice depends on the process requirement^15,16^. However, in slag valorization, using fresh fluxes increases the process costs and can negate its environmental benefits because they have to be mined and transported which emit additional CO_2_^15,17^. Furthermore, slags are typically valorized separately, losing the potential benefits of combining slags with complementary compositions.

Combining slags which have a distinct composition and properties^11,13^ potentially results in mixtures with melting and viscosity behavior suitable for smelting. Fayalite slag is acidic (silica-rich), whereas Waelz and Linz-Donawitz slags are basic (calcium-rich)^4,5,18^. The suitable basicity ratio (CaO/SiO_2_) for smelting is needed to be within the range of 0.9–1.0^19^. This basicity range is known to have a viable viscosity for slag and metal separation at 1450 °C process temperature^20^. By combining the calculation of different slag mixing ratios within a desirable basicity range with simulations and lab-scale experiments, it is possible to identify the feasible slag mixtures. This method offers a viable path for slag valorization and applies circular economy principles in the metallurgical industry^21^.

This study aims to create self-fluxing slag mixtures with the support of R to determine different slag mixing ratios that fall within the viable basicity range. Mixtures of Waelz, Linz-Donawitz and Fayalite slags were simulated using FactSage 8.2 and validated with laboratory-scale experiments. To further support the environmental susceptibility of the process, Toxicity Characteristic Leaching Procedure (TCLP) tests were done on the smelting experiment product slag. Present manuscript contributes to clear advance via a deeper understanding of slag behavior by linking basicity and viscosity to interfacial tension and by constructing an isoviscosity-ternary phase diagram of CaO-SiO_2_-Al_2_O_3_. The diagram provides a direct visual link between the viscosity values of the composition within the liquidus region. Detailed hydrodynamics was studied and found to be affected by the magnesium oxide (MgO) content. The current methodology gives a more comprehensive approach for designing self-fluxing slag mixtures that is feasible for smelting. This systematic approach bridges the gap between metallurgy and sustainability by applying circular economy principles.

## Materials, methods, and experimental

### Feed and product slag characterization

X-ray computed tomography (XCT) was used to inspect the internal structure and analyze the mixtures smelting viability. For elemental analysis of both metal and slag, Micro-XRF was used. X-ray diffraction (XRD) was employed to determine the phases of the slag products. The Waelz (W) and Linz-Donawitz (LD) slags were characterized in previous studies^4,20^. Fused bead Wavelength-dispersive X-ray fluorescence spectrometry (WD-XRF) was done to determine the oxide composition of the Fayalite slag. The XRF measurement was performed on a PANalytical Axios mineral spectrometer with a rhodium (Rh) tube. The data was collected using the manufacturer’s fully calibrated WROXI program, ensuring all elements were within the specified calibration range.

### Thermodynamic calculation and modeling

R open-source software was used to determine the suitable mixing of the slags to achieve a basicity ratio (CaO/SiO_2_) of 0.9–1.0^19^. Ternary plot was generated with the slags as the ternary axis (LD-W-F). Ten mixtures were identified across the ternary plot to study the trends and information to scale-up. Smelting of the identified mixtures was simulated using thermodynamic modeling (FactSage 8.2). The FToxid and FactPS databases were utilized in phase diagram module at 1450 °C. For visualization and to compare the feed slag compositions, only the normalized composition of CaO-SiO_2_-Al_2_O_3_ were considered. However, all components were considered in the FactSage equilibrium calculation. Furthermore, the FToxid, FactPS, FTstel, and FTmisc were used for equilibrium modeling at 1450 °C. Through this simulation, the amount of C needed for reduction of Fe, Mn, V, Cu, and Zn was calculated. The aim of the smelting process is to recover the Fe, Mn, Cu, and V in the metal phase; to recover the Zn in the gas phase; and to produce a stabilize slag product. The compositions falling within the liquidus region of the ternary phase diagram were determined. Using FactSage viscosity module, at 1450 °C, the viscosity values of different slag compositions were approximated. The calculated viscosities was visualized as a function of composition applying heat mapping and plotting the iso-viscosity lines, to demonstrate how compositional changes affect viscosity values^21^.

### Furnace experiments

#### Tube furnace smelting (20 g scale)

**Fig. 1:**
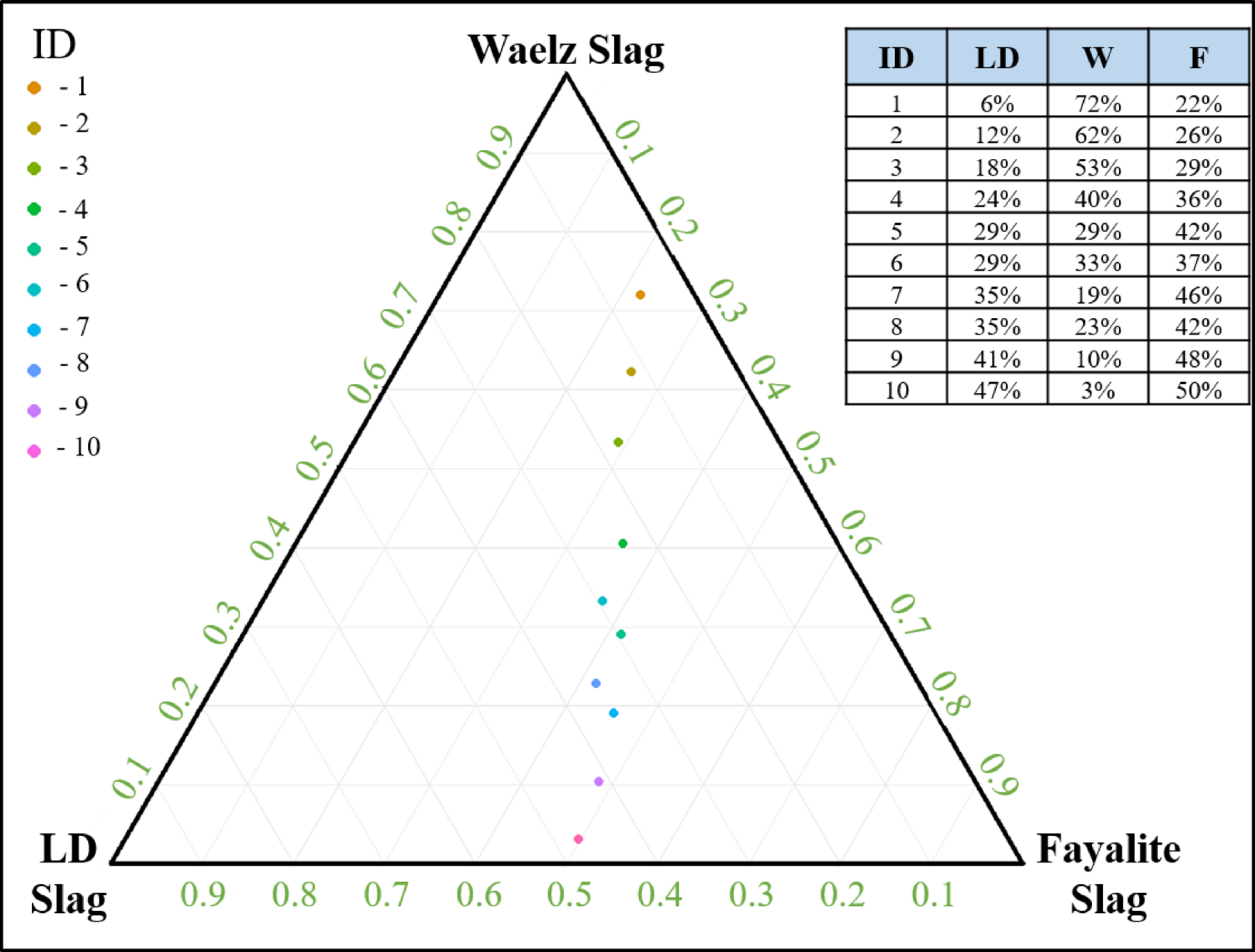
Feasible Slag Composition of LD, Waelz, and Fayalite with basicity CaO/SiO_2_ = 0.9–1.0 (created using R open source).

Twenty-gram scale smelting experiments were performed for each determined composition. The best performing composition was scaled to a 2 kg smelting experiment. The criteria for evaluation to scale up were the volume of the biggest metal nugget recovered from each mixture. Multiple experiments were conducted in a modified tube furnace (Nabertherm). From the graphite rods (Zinn-Gießerei-Göhler in Freital, Saxony, Germany), the crucibles were manufactured in-house with a height of 45 mm, 30 mm diameter and a 2 mm wall thickness. A graphite crucible was chosen to promote a reducing environment during the smelting experiment. The charges were milled accordingly, < 400 μm for petcoke, W and F slags, and < 1 mm for LD slag. The furnace was heated to 1450 °C with a 180 °C/h ramp during the experiment. After the furnace reached 1450 °C (process temperature), the crucibles were positioned into the hot area of the furnace. Temperature about 10 mm next to the crucibles was monitored with a type B thermocouple. The holding time of the process temperature was 4 h. After the holding time, the furnace was cooled down at 180 °C/h until room temperature. Finally, the crucibles and smelting products were collected for further characterization. The schematic diagram of the tube furnace experiment is shown in (Electronic Supplementary Material (ESM)) S-Figure 1.

#### Induction furnace smelting (2 kg scale)

**Fig. 2:**
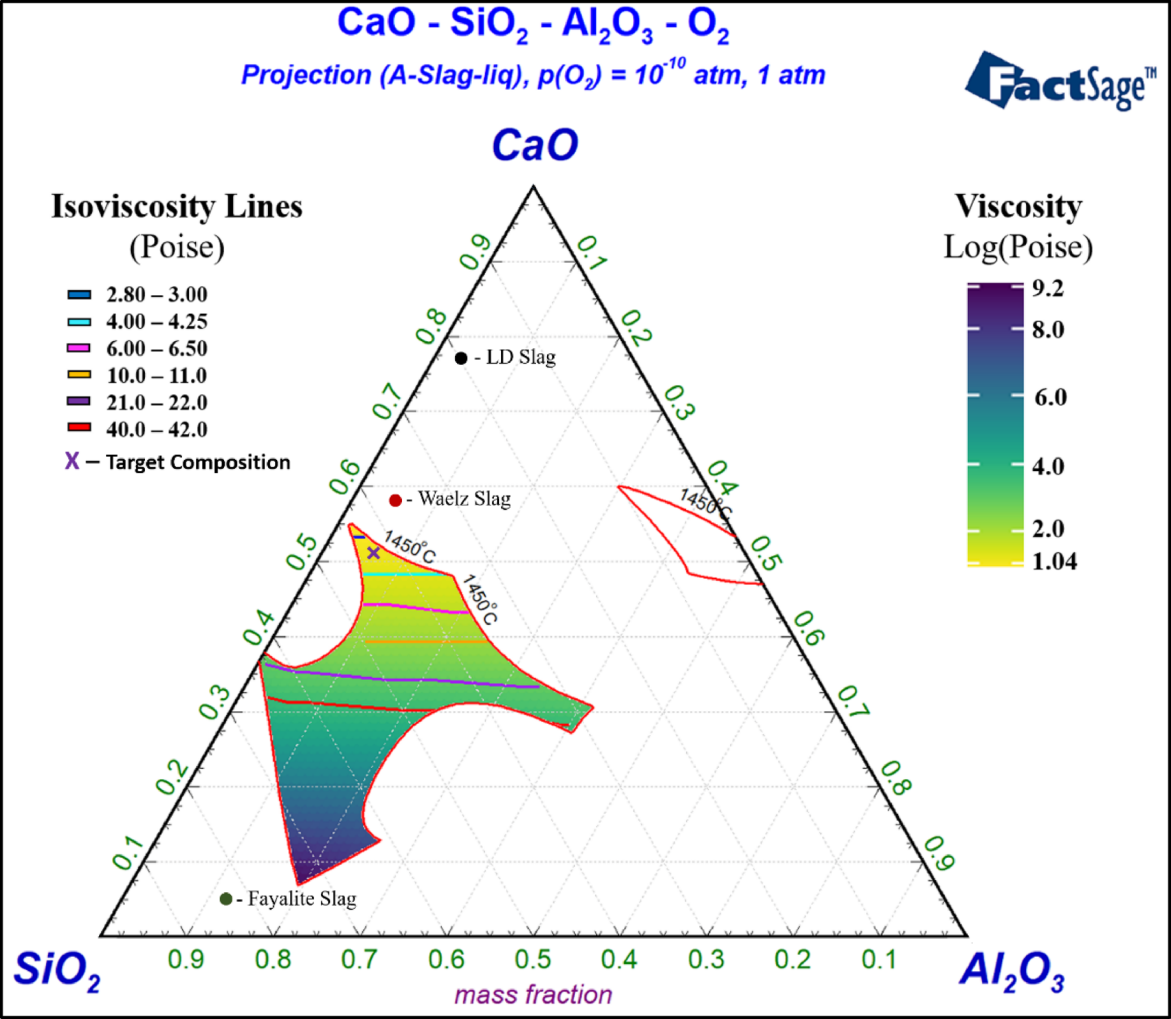
Ternary phase diagram of SiO_2_-CaO-Al_2_O_3_ of 1450 °C liquidous region with Viscosity Heat Map and Iso-Viscosity Lines. Feed slag composition and target composition plotting (created using FactSage 8.2 and R open source).

**Fig. 3:**
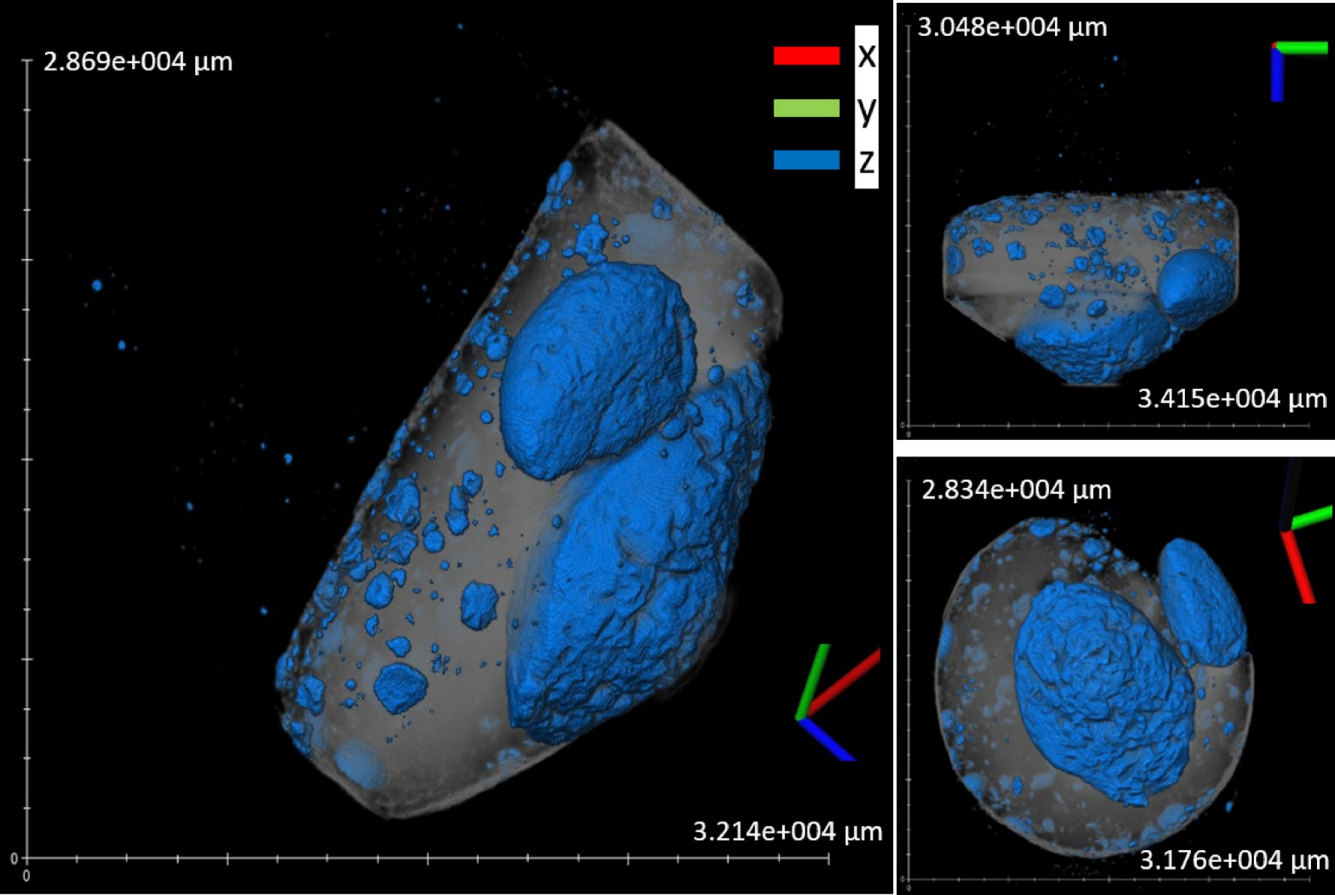
XCT images of smelting products from the tube furnace experiment of mixture ID 9: Gray regions indicate slag, while blue regions represent the metal.

The mixture with the highest metal nugget recovered in the 20 g scale experiment was scaled up to a 2 kg scale smelting experiment. The charges for this experiment, petcoke, F slag, and W slag, were prepared with particle sizes less than 10 mm, while LD slag less than 1 mm. The charges were mixed in a clay graphite crucible (Mammut-Wetro Schmelztiegelwerk GmbH – No. A15, mouth diameter: 180 mm, stage diameter: 120 mm, height: 230 mm). The experiment temperature was 1450 °C at an induction furnace (EMA-TEC, up to 35 kW, 3–7.2 kHz). The smelting experiment was carried out for 3 h at operating temperature (1 h – temperature homogenization; 1 h – argon (Ar) lancing for turbulence mixing; 1 h – settling). The schematic diagram of the scale-up experiment is shown in ESM S-Figure 2. The temperature profile of the 2 kg smelting experiment is shown in ESM S-Figure 3.

### Post smelting products (slags and metals) characterization

#### X-ray computed tomography

The bulk of the smelting products of the tube furnace experiment was initially scanned with XCT to study the internal structure of slag and metal droplets. The 3D scans were carried out using TESCAN CoreTOM (TESCAN - XRE, Ghent, Belgium). After the bulk scan, the crucible was broken to recover the large metal droplets. These isolated metal droplets were weighed and re-scanned to calculate the metal density. Through these methods, the total mass of the metal can be calculated. The tube furnace experiment products and the isolated metal droplets samples were imaged using a voltage of 160 kV, a power of 30 W, a 1 mm thick stainless-steel filter and a voxel size of 68 μm. The exposure time varied from 1150 ms to 1300 ms, depending on the sample. The metal droplet from the 2 kg scale experiment was scanned in high resolution with a voxel size of 16 μm with an exposure time of 900 ms per projection to allow the visualization of the pores inside the metal. The 2 kg scale sample was scanned using a voltage of 180 kV, a power of 40 W, a 1 mm thick stainless-steel filter, a voxel size of 160 μm, with exposure time of 850 ms per projection. The data reconstructions were carried out using Panthera^™^ (Tescan proprietary software, version 1.3.1) and the reconstructed images were processed and visualized using Avizo software (v9.3, Thermo Fisher Scientific, Waltham, MA, USA).

#### Elemental and phases analysis via micro X-ray fluorescence and X-ray diffraction

For bulk elemental analysis of both metals and slags, Micro-XRF (M4 Tornado, Bruker) was used. Phase composition was determined by XRD on a PANalytical Empyrean diffractometer equipped with a Co-Tube and a PIXcel 3D medipix 1 × 1 area detector. ICDD PDF-4 + database was used for identification. For quantitative analysis, Rietveld method and Profex/BGMN v.4.1 software was used^22^.

### Roasting of slag product from 2 kg scale smelting experiment

Slag was recovered and milled to less than 400 μm, then dried at 105 ± 5 °C. Around 210 g of dried slag was loaded into a mullite flatbed crucible (length: 16 cm; width: 10.5 cm; height: 3 cm), then subjected to 900 °C for 2 h in the presence of air (Linn High Therm LM 512.27). Roasting was done in duplicates. The phase composition of pre- and post-roasting slags was determined by XRD.

### TCLP test for slag from 2 kg smelting experiment (pre- and post-roasting)

The TCLP testing employed by this study followed US EPA Method 1311^23^. 1:20 solid-to-liquid ratio of sample and liquid extractant was charged into a 2 L borosilicate extraction vessel with the determined extraction fluid (solid-to-liquid ratio of 1:20), rotated end-over-end for 18 ± 1 h. The slurry was filtered through a 0.7 μm pore-sized glass fiber filter (Carl Roth GmbH + Co. KG). Lastly, 1 N HNO_3_ was added to acidify the solution to pH < 2, and analyzed with ICP-OES/MS. Similar procedure has been demonstrated in our previous study about TCLP testing for smelting slags^20^.

## Results and Discussion

### Characterization of slag feed

Table 1 shows the oxide compositions of W, LD, and F slags. In terms of basicity, the LD slag is the most basic (CaO/SiO_2_ = 3.75), followed by the W slag with an intermediate basicity (CaO/SiO_2_ = 1.57), with both slags containing high amount of calcium ferrite phase^4,20^. The F slag is the most acidic due to high silica (CaO/SiO_2_ = 0.07). All the feed slags major slag forming constituents are (2.23–42.26 wt% CaO), (10.00–33.92 wt% SiO_2_), (1.63–10.01 wt% MgO), and (1.50–4.86 wt% Al_2_O_3_), suggesting that blending them could produce a self-fluxing mixture.

**Table 1:** Oxide composition of feed slags. “ – ” - not measured.

Oxide Components	Wt%		
	LD Slag	W Slag	F Slag
*Al*_*2*_ *O*_*3*_	*1.58*	*1.50*	*4.86*
BaO	0.01	-	0.06
C	BDL	8.09	-
*CaO*	*42.26*	*15.67*	*2.23*
Cr_2_O_3_	0.42	-	0.08
CuO	-	-	0.97
Fe	-	9.26	-
FeO	-	0.60	52.12
Fe_2_O_3_	25.71	37.78	-
K_2_O	0.02	1.54	1.18
MgO	10.01	1.87	1.63
MnO	-	7.76	BDL
Mn_3_O_4_	3.66	0.00	0.12
Na_2_O	0.01	0.84	0.38
NiO	-	-	0.06
P_2_O_5_	0.47	-	0.13
PbO	-	-	0.13
*SiO*_*2*_	*11.28*	*10.00*	*33.92*
S	-	1.54	-
SO_3_	0.25	-	0.89
SrO	0.01	-	-
TiO_2_	1.31	-	0.36
V_2_O_5_	3.01	-	0.02
ZnO	-	3.54	0.79
ZrO_2_	-	-	0.01

### Thermodynamic calculation and modeling

Figure 1 shows the ternary diagram of the feed slags, with each corner of the triangle representing 100 wt% of one component and 0 wt% of the other two. Each point on the diagram represents a specific mixture of LD, W, and F slags that falls within the basicity range of (CaO/SiO_2_) of 0.9–0.99, which is within the viable range for smelting experiments^19^. Connecting these points reveals a line that can be referred to as the optimal composition line (OCL), representing the region of most favorable smelting conditions. The diagram illustrates the complex relationship between slag composition and basicity, which is essential for industrial applications. Equation 1 shows the normalization of proportions between W, LD, and F slags to have a valid ternary mixture. Equation 2 sets the condition that the ratio of the total CaO to total SiO_2_ falls within the range of 0.9–0.99. The intersection of these two equations forms a line in the ternary space representing the OCL. The data points are clustered toward the F slag region, suggesting that optimal compositions are achieved when F slag constitutes the majority of the blend. The best-performing blends are IDs 7 and 9, which will be discussed later. This method enables a plant operator to maintain a blend that falls along the OCL, providing operational flexibility.

1 $$\mathrm{W}+\mathrm{L}\mathrm{D}+\mathrm{F}=1$$

2 $$\frac{15.67\mathrm{W}+42.26\mathrm{L}\mathrm{D}+2.23\mathrm{F}}{10\mathrm{W}+11.28\mathrm{L}\mathrm{D}+33.92\mathrm{F}}=0.9-0.99$$

**Fig. 4:**
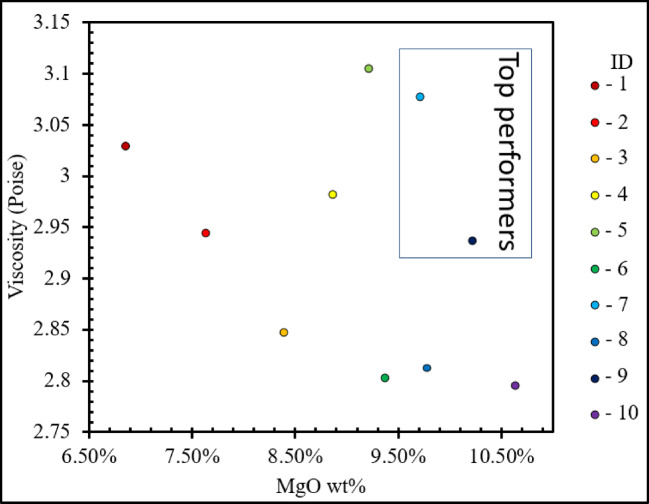
Viscosity (Poise) vs. FactSage MgO wt% of feed material.

ESM S-Figure 4 shows the isothermal lines of the SiO_2_-CaO-Al_2_O_3_ ternary phase diagram at 1450 °C with the location of 10 identified mixtures marked. Notably, all the identified mixtures selected along the OCL are within the 1450 °C liquidous region. ESM S-Tables 1, 2, 3, 4, 5, 6, 7, 8, 9 and 10 shows the predicted compositions of every mixture’s slag phase, metal phase, and gas phase by FactSage. Figure 2 shows the SiO_2_-CaO-Al_2_O_3_ ternary phase diagram at 1450 °C with viscosity values for different compositions within the liquidus region. This specific temperature and compositional range were selected based on previous study showing the feasibility of Waelz slag valorization^20^. Further, it illustrates how the slag composition affects the viscosity; further, previous literature shows that FactSage viscosity results are similar to the experimental values^21^. It shows the position of W, LD, and F slag’s normalized SiO_2_-CaO-Al_2_O_3_ values, along with the target composition. Notably, the heat map uses log(viscosity) to enhance the gradient between viscosity values. The warmer colors (yellow) have lower viscosity values (more fluid), while cooler colors (blue) denote higher viscosity. The contour lines show the viscosity values of slag composition within the specified viscosity range. Moreover, the feed slag’s initial position implies that fluxing is required to ensure a molten slag. However, by blending LD, W, and F slags, the target composition is achievable. Overall, it implies that Figs. 1 and 2 could serve as a “roadmap” for smelting, showing how different mixtures affect viscosity, leading to hitting the sweet spot for efficient smelting operation.

### Furnace experiments

#### XCT Characterization of bulk products (tube furnace experiment)

XCT results allowed the examination of the internal structure of the bulk products. The difference in gray values indicates differences in density, where brighter areas represent the metals and darker regions represent the lighter oxide slag. Reconstructing this allows the calculation of the internal structure and the total volume of metal. This analysis provides insights into the metal separation and agglomeration of each mixture. The volume of the metal nugget in the bulk measurement of every mixture closely matched the measurements of the separated individual metal nuggets, with a difference of 0.1–1.5%.

**Fig. 5:**
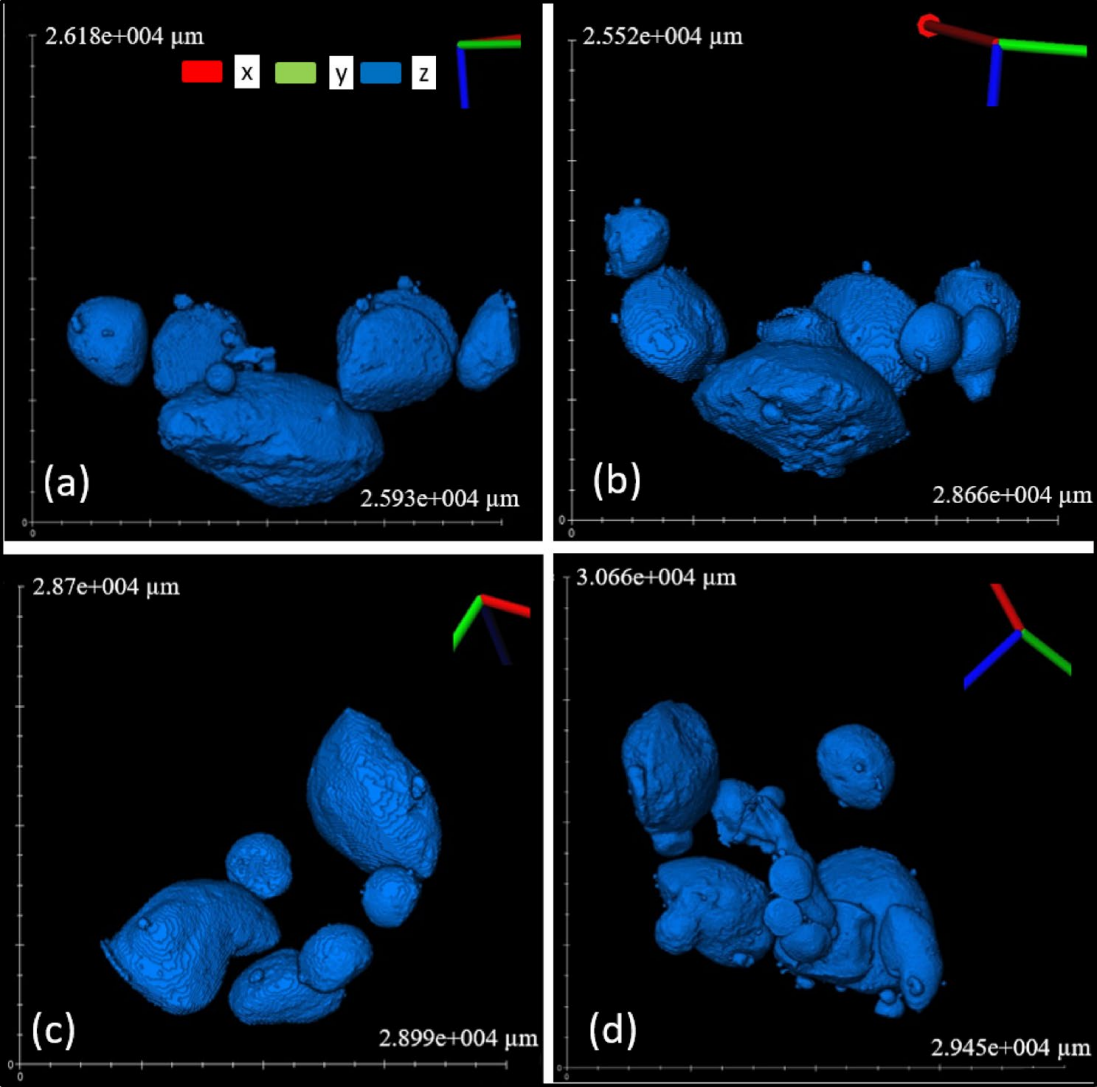
XCT analysis of metal droplets bigger than 1.78 × 10^10^ µm^3^ (a) ID 5, (b) ID 6, (c) ID 10, (d) ID 4.

**Fig. 6:**
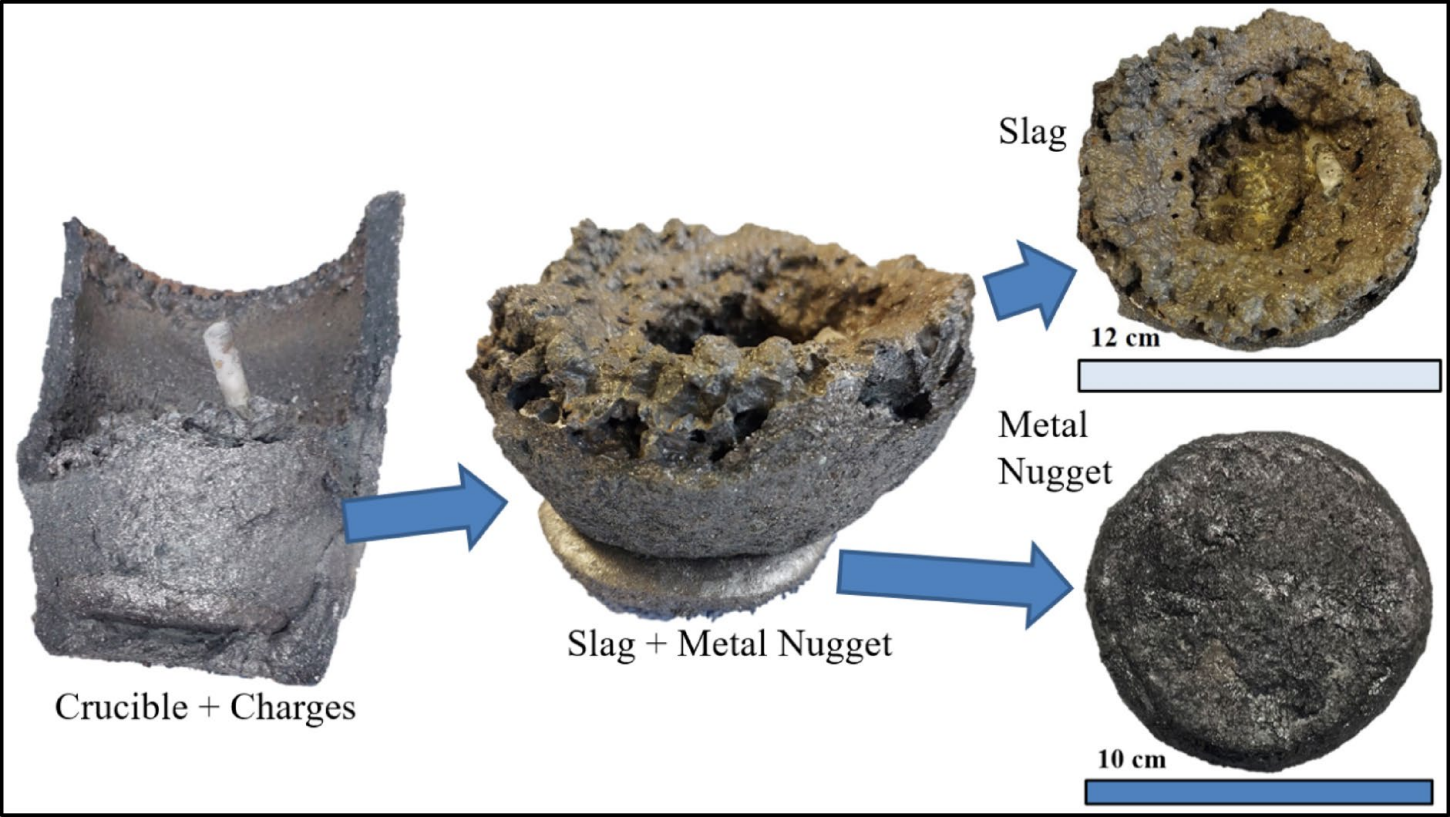
Products of induction furnace smelting.

**Fig. 7:**
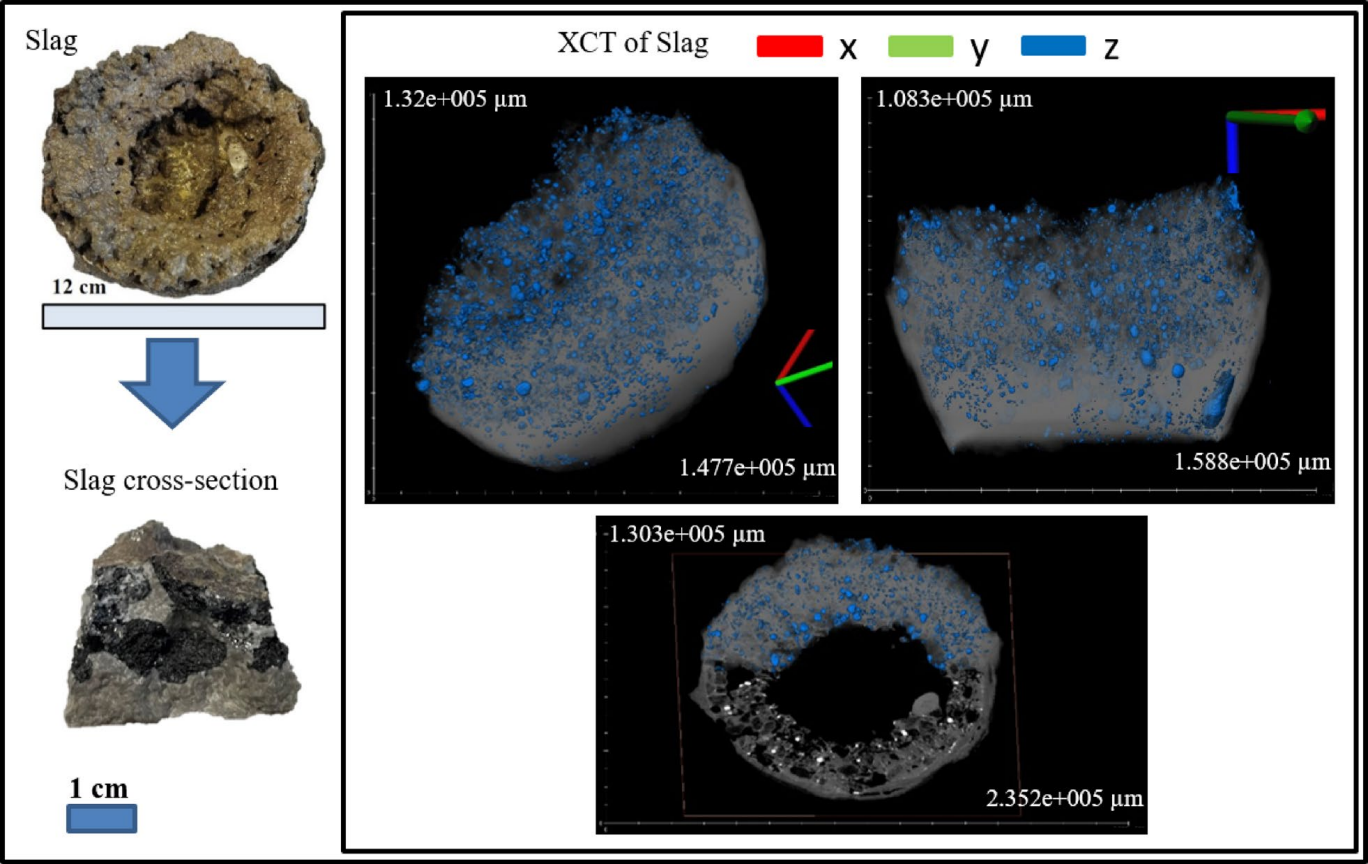
Actual photograph and X-ray computed tomography (XCT) image of slag (gray) and metal droplets (blue).

**Fig. 8:**
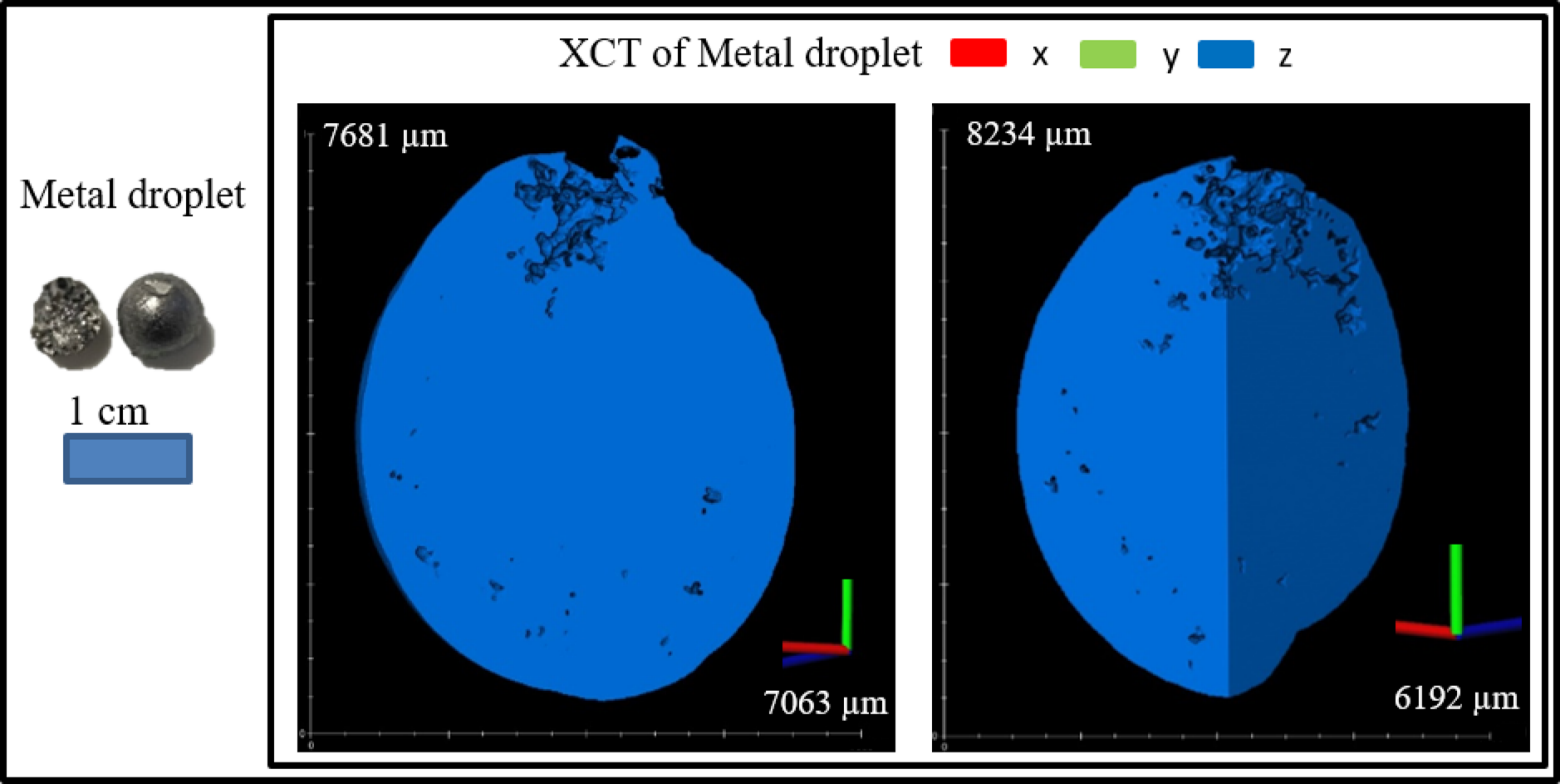
Internal structure and pores of metal droplets.

**Fig. 9:**
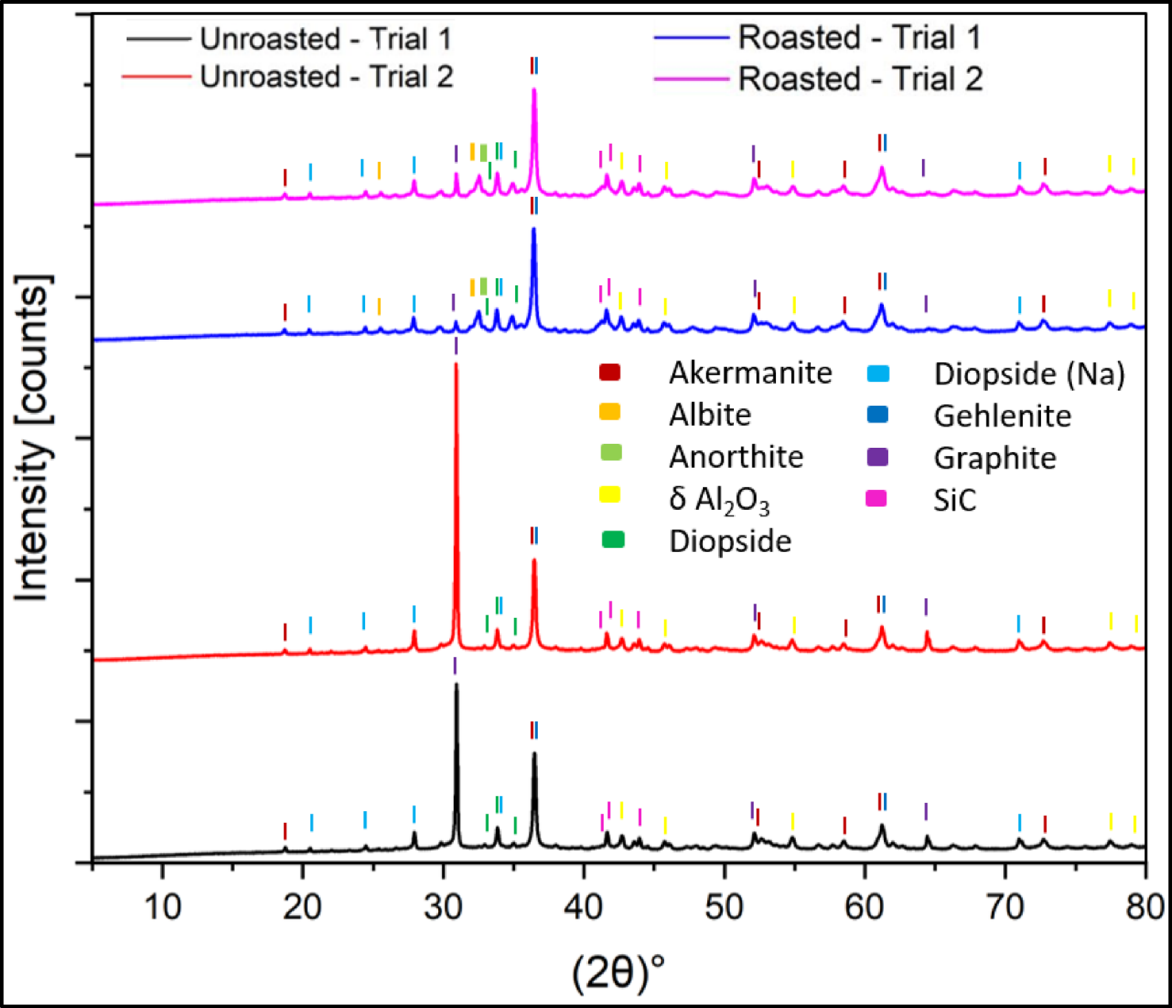
XRD plot of pre- and post-roasting product of scale-up smelting.

**Fig. 10:**
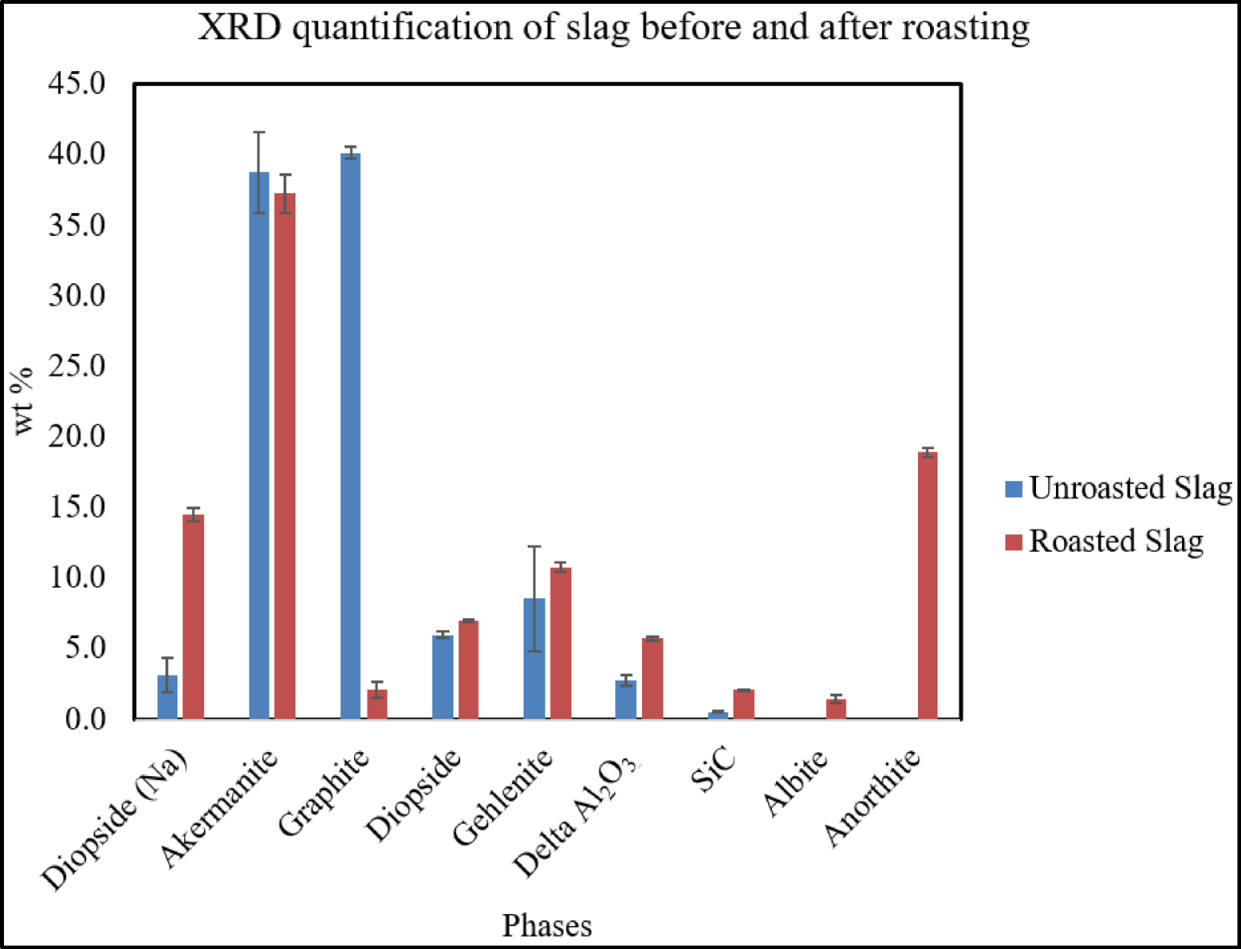
XRD quantification of slag before and after roasting.

Figure 3 shows the bulk XCT images of the best-performing mixture (ID 9), which has the biggest metal nugget volume of 0.75 cm^3^, a weight of 5.21 g, and thus with a density of 6.97 g/cm^3^. This mixture was chosen for scaling up to a 2 kg smelting experiment. A summary of the recovered metal nugget volume, the recovered metal nugget mass, the density of metal, the total metal volume, the actual metal mass, the volume of the biggest metal nugget, the sum of the five biggest nugget in each mixture, the basicity ratio, the predicted metal yield from thermodynamic modeling of FactSage, the FactSage oxygen partial pressure and the metal mass relative error for all mixtures is provided in Table 2. Additional XCT images for the other mixtures are provided in (ESM S-Figs.  4, 5, 6, 7, 8, 9, 10, 11, 12 and 13).

**Table 2:** All identified mixtures: Recovered metal nugget volume, density, total metal volume, total actual metal mass, basicity ratio, FactSage metal mass, and metal mass relative error. RMNV – Recovered Metal Nugget Volume; RMNM – Recovered Metal Nugget Mass; D – Density; TMV – Total Metal Volume; VBMN – Volume of Biggest Metal Nugget; SFBN – Sum of Five Biggest Nugget; TAMM – Total Actual Metal Mass; FBR- Feed Basicity Index; FSMM – FactSage Metal Mass; FOPP – FactSage Oxygen Partial Pressure; MM – Metal Mass.

	ID									
	1	2	3	4	5	6	7	8	9	10
**RMNV (cm** ^**3**^ **)**	0.03	0.03	0.36	0.27	0.53	0.52	0.74	0.21	0.75	0.32
**RMNM (g)**	0.24	0.18	2.53	1.91	3.73	3.6	5.19	1.48	5.21	2.21
**D (g/cm** ^**3**^ **)**	7.50	7.2	7.03	7.07	7.04	6.92	7.01	7.05	6.97	6.91
**VBMN (cm** ^**3**^ **)**	0.07	0.05	0.36	0.40	0.53	0.52	0.74	0.22	0.75	0.38
**SFBN (cm** ^**3**^ **)**	0.19	0.15	0.87	0.96	0.84	0.91	0.90	0.78	0.93	0.87
**TMV (cm** ^**3**^ **)**	1.21	1.02	1.06	1.11	1.00	1.11	0.99	1.01	0.99	0.98
**TAMM (g)**	9.08	7.34	7.45	7.85	7.04	7.68	6.94	7.12	6.90	6.77
**FBI (CaO/SiO**_**2**_ **)**	0.95	0.96	0.99	0.93	0.91	0.99	0.92	0.98	0.94	0.99
**FSMM (g)**	8.46	8.22	8.00	8.42	7.50	7.40	7.22	7.22	6.86	6.70
**FOPP (atm)**	1.22 × 10^− 16^	1.23 × 10^− 16^	1.24 × 10^− 16^	1.26 × 10^− 16^	1.28 × 10^− 16^	1.27 × 10^− 16^	1.30 × 10^− 16^	1.29 × 10^− 16^	1.31 × 10^− 16^	1.33 × 10^− 16^
**MM Difference (%)**	7.07	11.3	7.12	7.01	6.33	3.71	3.95	1.39	0.58	1.03

##### Micro-XRF of metals and slags for elemental analysis and material balance (tube furnace experiment)

The composition of metals and slags from each mixture was presented in ESM S-Tables 11 and 12. Moreover, the elemental balance, as presented in ESM S-Tables 13 and 14, shows the overall distribution of elements. The apparent recoveries are affected by several sources of analytical error. In particular, the recovery of some elements shows to exceed 100% because pores formed within the metal droplets increase the measured volume, which overestimates the total metal mass. A further complication is the blur effect observed during the XCT analysis, which can distort the image and lead to inaccurate quantification. Furthermore, even minor errors in the instrumentation have a disproportionately large impact, since the concentrations of many elements are extremely low; therefore, small uncertainties translate into large errors. Despite these challenges, the major element distribution between metal and slag of the products is consistent across all mixtures, as shown in Table 3. Furthermore, Zn was not detected in both the metal and slag, implying its complete fuming.

**Table 3:** Elemental distribution of the products.

ID	Phase	Al	Ca	Cu	Fe	Mg	Mn	Na	*P*	S	Si	Ti	V
1	Metal	2	0	99	100	0	82	0	96	4	24	0	98
	Slag	98	100	1	0	100	18	0	4	96	76	100	2
2	Metal	1	0	100	100	0	92	0	95	4	14	43	99
	Slag	99	100	0	0	100	8	0	5	96	86	57	1
3	Metal	1	0	99	100	0	86	100	93	3	16	33	99
	Slag	99	100	1	0	100	14	0	7	97	84	67	1
4	Metal	1	0	99	100	0	82	100	100	4	15	35	99
	Slag	99	100	1	0	100	18	0	0	96	85	65	1
5	Metal	1	0	99	100	0	87	100	100	6	18	35	99
	Slag	99	100	1	0	100	13	0	0	94	82	65	1
6	Metal	1	0	99	100	0	80	100	100	6	14	30	99
	Slag	99	100	1	0	100	20	0	0	94	86	70	1
7	Metal	1	0	100	100	0	85	100	100	9	14	39	98
	Slag	99	100	0	0	100	15	0	0	91	86	61	2
8	Metal	1	0	100	100	0	76	100	100	9	8	22	99
	Slag	99	100	0	0	100	24	0	0	91	92	78	1
9	Metal	1	0	99	100	0	81	0	100	12	9	27	98
	Slag	99	100	1	0	100	19	0	0	88	91	73	2
10	Metal	0	0	100	100	0	86	100	100	14	8	20	98
	Slag	100	100	0	0	100	14	0	0	86	92	80	2

##### Impact of different slag ratios on reduction efficiency (tube furnace experiment)

FactSage modeling shows that all slag mixtures tested fall within the recommended basicity range of 0.9–1.0, and all mixtures had similar Al_2_O_3_ contents of 6–7 wt%. Utilizing the FactSage reaction module using the FactPS database at 1450 °C, the calculations show that the fayalite reduction (Eq. 3) has a Gibbs free energy of −214 kJ, whereas for calcium ferrite reduction (Eq. 4) has a Gibbs free energy of −389 kJ. This indicated that the reduction of iron bound to CaO is more favorable than the reduction of iron bound to SiO_2_. With excess carbon in the charge and the use of graphite crucibles, the overall parameters were at very low oxygen partial pressure (pO_2_), favorably promoting the Fe reduction. This Fe reduction has a greater effect for calcium ferrite, which generates three moles of CO gas compared to only two moles for fayalite. Thermodynamically, Fig. 5.54a of Slag Atlas^24^; supports that FeO activity is higher in the CaO-FeO region than in the SiO_2_-FeO region, implying that Fe in calcium ferrite is readily available for the reduction than in fayalite. Furthermore, the equilibrium module in FactSage at 1450 °C, shown in ESM S-Figure 14, implies that increasing CaO decreases the Fe content in the slag more effectively than increasing SiO_2_ content. The current study shows that almost complete Fe reduction occurred in all mixtures, as shown by a small relative error in metal mass (Table 2). Even the least effective mixtures (IDs 1 and 2) achieved very high Fe reduction. Still, due to high slag viscosity and low surface tension droplet coalescence was hindered, resulting in dispersed metal droplets. Moreover, the FactSage viscosity module at 1450 °C implies that within the recommended basicity range of 0.9–1.0, viscosity values decrease proportionally with increasing basicity, as shown in ESM S-Figure 15.

3 $${\mathrm{F}\mathrm{e}}_{2}{\mathrm{S}\mathrm{i}\mathrm{O}}_{4}+2\mathrm{C}2\mathrm{F}\mathrm{e}+{\mathrm{S}\mathrm{i}\mathrm{O}}_{2}+2\mathrm{C}\mathrm{O}{\Delta}\mathrm{G}=-214\mathrm{k}\mathrm{J}$$

4 $$\mathrm{C}\mathrm{a}{\mathrm{F}\mathrm{e}}_{2}{\mathrm{O}}_{4}+3\mathrm{C}2\mathrm{F}\mathrm{e}+\mathrm{C}\mathrm{a}\mathrm{O}+3\mathrm{C}\mathrm{O}{\Delta}\mathrm{G}=-389\mathrm{k}\mathrm{J}$$

##### Effects of viscosity and MgO content on metal nugget formation (tube furnace experiment)

Table 2 shows that IDs 7 and 9 were the best performers, since they had the largest metal nuggets. The next top performers were mixtures IDs 5 and 6, followed by IDs 3, 4, 8, and 10. Interestingly, mixtures with IDs 1 and 2 have the poorest performance, producing fine and dispersed metal particles trapped in the slag, as shown in ESM S-Figs. 5 and 6. These results show the interplay between viscosity, surface tension, and interfacial tension of metal nugget formation. In the Al_2_O_3_-SiO_2_-CaO slag system, MgO addition has been found to increase the surface tension^25,26^. Girifalco and Good’s equation shows that increasing surface tension leads to a corresponding increase in interfacial tension^27,28^. Higher interfacial tension favors the formation of larger nuggets. Interfacial tension was not directly measure in this study but inferred from MgO content based on established empirical correlations^25–28^. As the system progresses through a thermodynamically favorable state, nugget formation proceeds through a cyclic sequence of droplet formation, flattening, the generation of smaller droplets, and subsequent coalescence^29^. Consequently, the nuggets tend to coalesce more readily under the relatively high interfacial tension, low slag viscosity, and small relative velocities of colliding droplets^30^. Also, at low viscosity the mass transfer rates are higher and thus reaction kinetics is increased. This is observed with IDs 7 and 9, as shown in Fig. 3 and ESM S-Figure 11. In addition, low interfacial tension promotes the flattening of molten metal droplets in order to maximize their surface area; once a critical flattening threshold is reached, the metal disintegrates into smaller, spherical droplets^31^. This behavior was evident in the present work for the low‑MgO mixtures (IDs 1 and 2), as illustrated in ESM S‑Figs. 4 and 5.

Figure 4 shows that MgO content, which is related to interfacial tension, is correlated with viscosity that affects metal‑nugget size and position. ID 5 has a relatively high MgO level, but the amount is still too low for strong bigger droplet coalescence. Its melt also has the highest viscosity, which hinders particle settling; as observed, a few nuggets are just above the slag region. We have also reported such a hindrance in metal nugget formation due to high slag viscosity^20^. ID 6 contains more MgO and has much lower viscosity. Its biggest metal nugget produced is smaller compared to ID 5, where higher viscosity promoted nugget retention. For droplets of similar size, those in ID 5 remained trapped in the slag due to higher viscosity, while in ID 6 they settled at the bottom flatter and more easily fragmented, as shown in Fig. 5a and b. IDs 8 and 10 have MgO contents similar to IDs 7 and 9, but their low viscosities might prevents the formation of larger stable nuggets, as shown in Fig. 5c. IDs 3 and 4 show higher MgO than IDs 1 and 2, producing larger nuggets than those samples, yet their nuggets remain smaller than those in IDs 7 and 9, as shown in Fig. 5d. Thus, increasing MgO can promote nugget growth, but only when the melt viscosity stays within an optimal range.

#### Scale-up experiment (induction furnace)

The top three mixtures in descending order with respect to the total metal content were ID 4, ID 3, and ID 6; while for the sum of the five biggest nuggets were ID 4, ID 9, and ID 6; and the biggest nugget were ID 9, ID 7, and ID 5. These rankings indicate that a mixture with high total metal even though has high collision probability does not necessarily mean to be the optimized mixture as dispersed metal is difficult to recover (ESM S-Figs. 5 and 8, and 9). The sum of the five largest nuggets shows that big nuggets are formed but their spatial location is critical for recovery. ID 4 has the highest sum however most nuggets are located in the middle section, making recovery difficult (ESM S-Figure 8). ID 9 and ID 7 has the biggest nugget formed in the bottom implying ease of recovery upon scale-up (Fig. 3 and ESM S-Figure 11). ID 7’s second biggest nugget is located near the middle section, whereas ID 9’s second biggest nuggets is near the bottom; thus, ID 9 is identified as the most optimized mixture. In all the tube-furnace experiments, ID 9 produced the largest nugget and was therefore scaled to a 2 kg induction-furnace smelting; the products of the smelting are shown in Fig. 6. The feed mixture is composed of 0.82 kg LD slag, 0.21 kg WS slag, 0.97 kg FS slag, and 0.26 kg petcoke (total 2.26 kg). The scale-up smelting produced a metal nugget of 700 g (~ 31 wt% of the feed) with composition Fe – 86.53 wt%, C – 1.65 wt%, Mn – 2.28 wt%, Cu – 1.09 wt%, V – 1.81 wt%, S – 0.44 wt%, Si − 6.39 wt%. The recovery of these elements in the metal nugget is: Fe – 98%, Mn – 46%, Cu – 96%, V – 91%, S – 41%, Si – 22%. The metal nugget can be considered a vanadium-bearing pig iron which has a composition between FeSi10 and cast iron; however, with its vanadium content, it can serve as a complex alloy addition, it only requires a further desulfurization step. Studies have also shown the possible vanadium extraction route of vanadium-containing pig iron and its effects in ductile cast iron^32–34^. A comparison table of metal nugget recovered to different standard is shown in ESM S-Table 15.

The scale-up operated at the same temperature and viscosity as the tube-furnace experiment. In ID 9, FactSage predicted that the metal yield is 34% of feed weight. However, the tube-furnace experiment yielded a nugget of ~ 23 wt% of the feed in the bottom and has Fe, Mn, Cu, and V recovery of 77%, 46%, 76%, and 45%, respectively. Moreover, the condensates at the upper cooling head of the furnace were measured using XRF and confirmed the presence of Zn. The rest of the metal is dispersed and trapped in the slag (Fig. 3). In contrast, the scale-up experiment has much higher recovery despite a 1 h shorter total processing time. This improvement is attributed to turbulence mixing by Ar lancing which increases the collision frequency and coalescence as well as mass transfer rates which align with the literature^5^. This resulted in producing larger nuggets, thus resulting in higher settling velocity leading to more efficient slag-metal separation and higher metal yield. Overall, the scale-up supports the process-design workflow of this study, (1) mixture selection in R, (2) phase-diagram, (3) calculation of carbon needed and (4) viscosity calculations using FactSage.

Figure 7 shows the entrapment of metal droplets within the slag, while Fig. 8 further illustrates that many of these droplets contain pores, which inflates the metal apparent volume. The mass of the recovered droplet was 1.06 g with a volume of 0.154 cm^3^ equating to a density of 6.88 g/cm^3^. XCT calculations without correction show that the total volume of the droplets was 10.3 cm^3^; thus, the total uncorrected weight of trap droplets was 70.86 g (3.14 wt% of feed). The presence of internal voids directly accounts for the slight overestimation in overall metal recovery, which is: Fe − 108%, Mn – 50%, Cu – 106%, V – 100%, S – 45%, Si – 24%, as a portion of the metal is not solid metal but empty space. From Table 2, the density of ID 9 droplet was 6.97 g/cm^3^ then by applying this density the number of pores in the trap droplet was 1.25% with respect to volume. Assuming that the pore to volume ratio is the same in all the trap droplets after correcting due to porosity effect, the total droplet volume decreases to 10.17 cm^3^ yielding a corrected mass of 70.88 g. Accordingly, the corrected overall recovery was Fe − 108%, Mn – 50%, Cu – 106%, V – 100%, S – 45%, Si – 24%. The oxide composition of the product slag is shown in Table 4.

**Table 4:** Oxide composition of product slag from induction furnace experiment.

Al_2_O_3_	CaO	MgO	Cr_2_O_3_	CuO	FeO	K_2_O	Mn_3_O_4_	NiO	SO_3_	SiO_2_	TiO_2_	V_2_O_5_
13	36.09	9.5	0.17	0.03	0.84	0.08	0.62	0.02	2.47	35.96	1.03	0.18

### Sulfur oxidation of slag product from 2 kg scale smelting experiment

The C/S analysis results of the slag show that roasting occurs since the sulfur content decreases from 0.61 wt% to 0.58 wt%, and the carbon content drops from 14.16 wt% to 0.86 wt%, as shown in ESM S-Table 16. Before roasting, the slag contained high graphite and low Na-diopside. After roasting, graphite was almost gone, and then new plagioclase phases appeared. Albite and anorthite were formed, while the amount of gehlenite and Na-diopside increases, as shown in Figs. 9 and 10. According to literature the typical formation of anorthite ranges from approximately 1100 °C to 1250 °C implying the effects of differences in composition and flux content^35^. Moreover, it was demonstrated that flux-assisted CaO-Al_2_O_3_-SiO_2_ system can crystallize anorthite at temperature as low as 1100 °C^36^. These phase changes implied important downstream applications of the roasted slag. According to the literature, blast furnace and other non-ferrous slags, in which roasted slag has a similar composition can be used in the building industry and geopolymer materials^37,38^. Further, the roasted slag contains around 19 wt% anorthite, which has favorable properties for porcelain applications. Anorthite has thermal expansion coefficient similar to glassy phase which makes it high thermal shock resistance, low thermal expansion coefficient, and makes it suitable for low-temperature glaze^35^. Although the roasted slag itself has not yet been evaluated in the construction industry or porcelain formulation, its phase composition of albite, anorthite, gehlenite, and Na-diopside suggests its applicability in the ceramics and construction industry. From a process-engineering perspective, the roasting step can be integrated directly with the smelting operation by tapping the slag into a separate furnace or container without allowing it to cool down. Since the process temperature is 1450 °C, it implies that when it’s tapped, the roasting process can proceed immediately by air blowing, eliminating the need for additional heating.

### TCLP test of slag products

Both the unroasted and roasted slag were benchmarked against different regulations to confirm if they meet environmental safety requirements. The United States Environmental Protection Agency (US EPA)^39^ standards, the United Kingdom’s Environmental Agency leaching threshold^40^, and Germany’s Ersatzbaustoffverordnung (EBV)^41^ for recycled material. In Table 5, the comparison of the extract of both slags with all the criteria is shown. The TCLP extracts of unroasted and roasted had a pH of 4.27 and 3.92, respectively. From the results of the fluid selection test, extraction fluid 1 was used for both slags. The unroasted slag qualifies for Germany’s recycled building material class 1 (RC – 1). However, the roasted slag does not qualify due to its vanadium extracts exceeding the acceptable limits. Nevertheless, it qualifies as blast furnace slag class 1 (HOS – 1). Roasting at high oxygen partial pressures converts both the sulfur and vanadium into more leachable phases. Roasting under air at 900 °C oxidizes the vanadium to higher valence, similar observation occurs in vanadium leaching of roasted steel slags^42^. The unroasted slag is below the threshold concentration of the regulated elements and compounds, such as, arsenic, cadmium, chromium, copper, molybdenum, nickel, lead, sulphates, vanadium, and zinc and thus falls within the limits set by the US, UK, and Germany. Both slags qualify as non-hazardous by the US EPA standards and both slags are acceptable for granular waste for construction applications by the UK standard. However, only the unroasted slag qualifies for the German EBV RC − 1 category, implying its applicability as a construction material. Therefore, for the US and UK based regulations, both slags are considered environmentally compatible for ceramics and construction applications, but for the German standard, only the unroasted slag qualifies as recycled construction material (RC – 1).

**Table 5:** Total dissolved metals in the extracts of pre- and post-roasting and regulatory limits. HOS 1 – Blast furnace slag class 1 (Hochofenstückschlacke der Klassen 1); RC 1 – Recycled building materials class 1 (Recycling-Baustoff der Klassen 1); NG – No Guidelines; BDL – Below Detection Limit (* - ppb; # - ppm): As – 0.1^#^; Cd – 0.2^#^; Cr – 0.1*; Cu – 0.1*; Mo – 0.1^#^; Ni – 0.1^#^; Pb – 0.1^#^; S – 0.2^#^; V – 0.1*; Zn − 0.1^#^.

Element	Pre-Roasting	Post-Roasting	Germany HOS − 1 (µg/L)*	Germany RC −1 (µg/L)*	US (mg/L)^#^	UK EA (mg/kg)
As	BDL	BDL	NG	NG	5.0^#^	25
Cd	BDL	BDL	NG	NG	1.0^#^	5
Cr	8.57*	53.8*	NG	150*	5.0^#^	70
Cu	57.6*	12.1*	NG	110*	NG	100
Mo	BDL	BDL	NG	55*	NG	30
Ni	BDL	BDL	NG	NG	NG	40
Pb	BDL	BDL	NG	NG	5.0^#^	50
SO_4_	101.50^#^	296.18^#^	1300^#^	600^#^	NG	50,000
V	18.7*	21.65^#^	NG	120*	NG	NG
Zn	BDL	BDL	NG	NG	NG	200

## Conclusion

The valorization process for classified feeds such as Linz-Donawitz, Fayalite, and Waelz slag has been shown to be feasible for producing a pig iron, zinc dust and a stabilized secondary slag, thereby demonstrating a practical circular‑economy approach. R was applied to systematically determine different slag mixing ratios that fall within the viable basicity range. Then, the smelting of the determined mixtures were simulated using FactSage 8.2 and validated with laboratory-scale experiments. The use of X‑ray computed tomography proved invaluable for identifying the formulations that generate the largest metallic nuggets—an indicator of the most feasible composition. However, future studies can use more criteria for evaluation including slag properties and economic consideration to further strengthen the optimization. Nevertheless, the current study shows the influence of interfacial tension and viscosity on the smelting process. The resulting pig iron nugget of the scale-up smelting recovered iron, copper, manganese and vanadium at approximately 98%, 96%, 46%, and 91%, respectively. Depending on its exact composition, this pig iron can be redirected to steel‑making. By toxicity characteristic leaching procedure, the unroasted slag was found to be suitable for construction‑material classifying it as non‑hazardous. Moreover, the presence of albite, anorthite, gehlenite, and Na-diopside in the roasted slag makes it valuable for ceramic applications. The best optimized mixture was found to be 41 wt% Linz-Donawitz and 10 wt% Waelz and 48 wt% Fayalite, the selection was based on the metal nugget formation and its spatial location which indicates efficient slag-metal separation with high metal yield. This composition falls within the optimal composition line shown in Fig. 1. Overall, this work provides a comprehensive understanding of Waelz, Linz-Donawitz, and Fayalite slag valorization applying circular‑economy‑aligned process, from feedstock management to end‑product application.

## Supplementary Information

Below is the link to the electronic supplementary material.

Supplementary Material 1

## Data Availability

The data is provided in the ESM in the details to support the findings.Additional metadata will be made available on a reasonable request to the corresponding authors.
