# Supplementary material for: Pyrometallurgical valorization of waelz, fayalite, and linz-donawitz slag mixtures
Source: Sci Rep. 2026 Mar 19;16:9539. doi: 10.1038/s41598-026-44763-3 (PMC13004828; doi:10.1038/s41598-026-44763-3)
Supplement: Supplementary file 1 — Supplementary Material 1 [file 41598_2026_44763_MOESM1_ESM.docx]

# Pyrometallurgical Valorization of Waelz, Fayalite, and Linz-Donawitz Slag Mixtures

Junnile L. Romero^1,2,*^, Volker Recksiek^1,3,*^, Rafaela Debastiani^1^, Md Naziat Hossain^1,3^, Ludwig Blenau^3^, Alexandros Charitos^3^, Ari O. Vaisanen^2^, Ajay B. Patil^1,2,*^

^1^Helmholtz Zentrum Dresden Rossendorf – Helmholtz Institute Freiberg for Resource Technology (HZDR-HIF), Chemnitzer Strasse 40, Freiberg, Germany.
^2^The University of Jyväskylä, Faculty of Science and Mathematics, Department of Chemistry, P.O. Box 35, Jyvaskyla, FI-40014, Finland.
^3^Technische Universität Bergakademie Freiberg (TUBAF), Institute of Nonferrous Metallurgy and Purest Materials (INEMET), Leipziger Straße 34, Freiberg, Germany.

^*^Correspondence: [a.patil@hzdr.de](mailto:a.patil@hzdr.de); [v.recksiek@hzdr.de](mailto:v.recksiek@hzdr.de); [j.romero@hzdr.de](mailto:j.romero@hzdr.de)

Keywords: circular-economy, Waelz slag, Fayalite Slag, LD Slag, smelting, pyrometallurgy


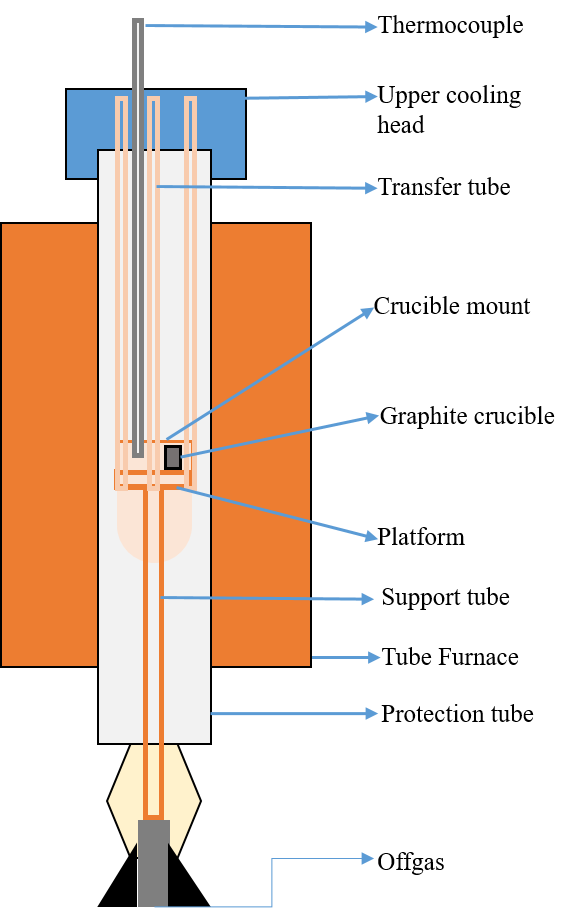


S-Figure 1 Tube furnace experiment


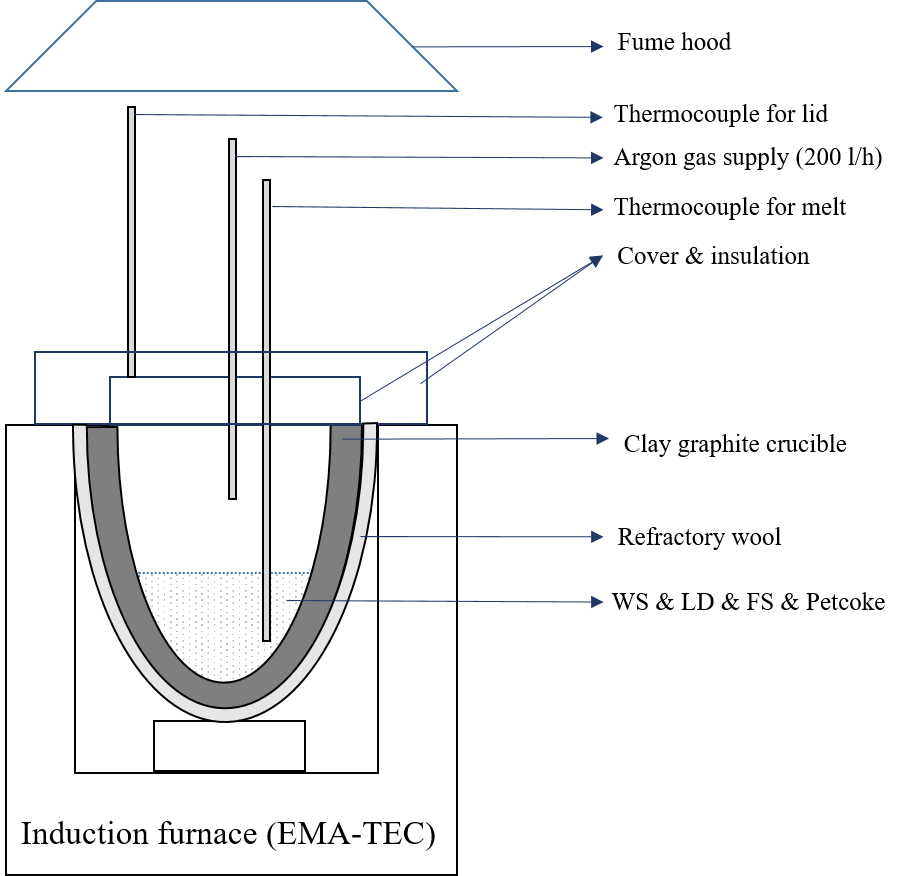


S-Figure 2 Induction furnace experiment

**
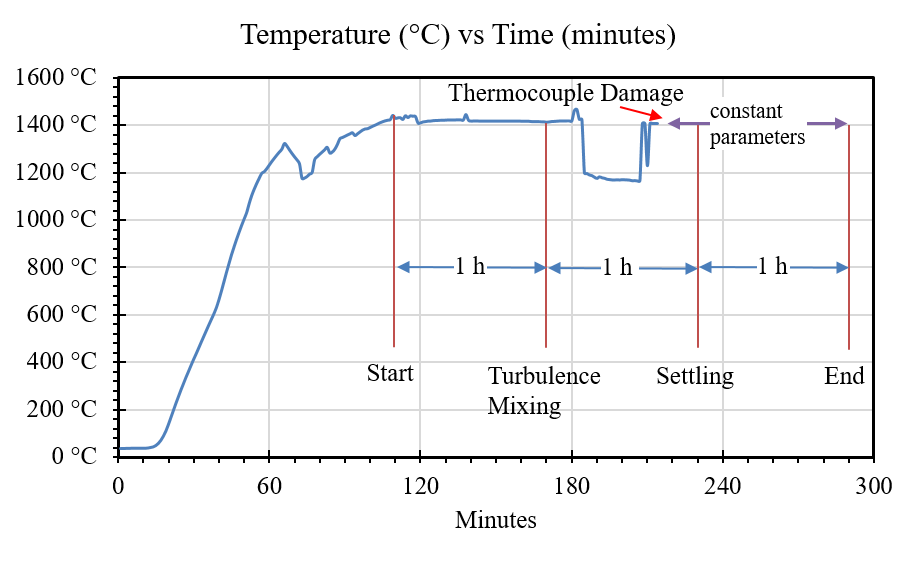
**

S-Figure 3 Temperature profile of 2-kg smelting experiment


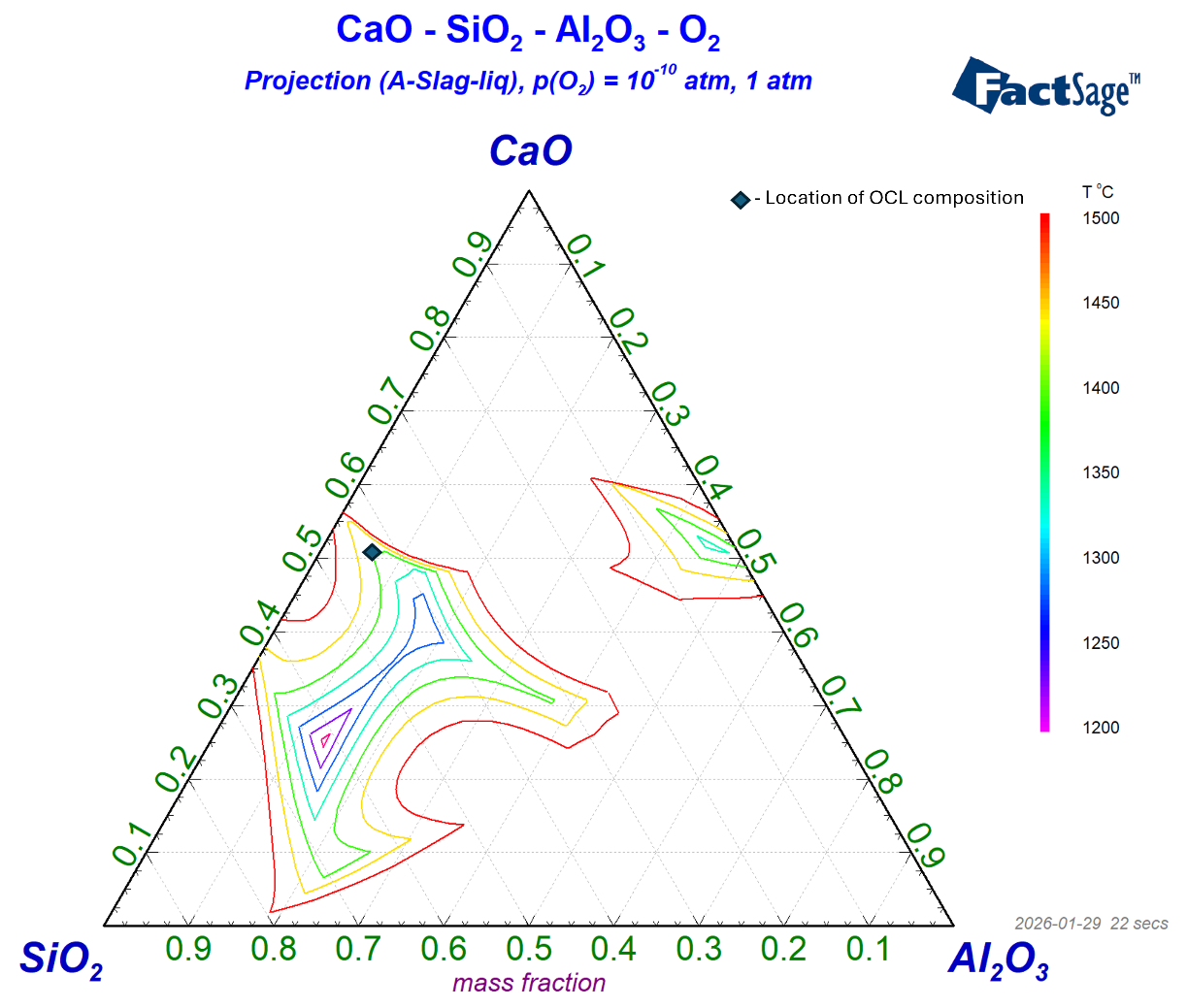


S-Figure 4 SiO_2_-CaO-Al_2_O_3_ Ternary Phase Diagram with isothermal lines location of identified mixtures

S-Table 1 FactSage expected distribution of ID 1 composition of metal phase, slag phase, and gas phase.

| ID 1 | | | | | | | |
| --- | --- | --- | --- | --- | --- | --- | --- |
| Metal (42.907 g) | | Slag 1 (33.826 g) | | Slag 2 (1.4618 g) | | Gas (1 atm) | |
| Phase | wt. % | Phase | wt. % | Phase | wt. % | Phase | Mass (a) |
| Al | 5.06E-04 | Na_2_O | 0.82559 | Na_2_O | 0.16028 | CO | 0.93961 |
| C | 4.5546 | K_2_O | 0.93238 | K_2_O | 1.69E-01 | Zn | 3.38E-02 |
| Cu | 0.37641 | Al_2_O_3_ | 5.2623 | Al_2_O_3_ | 6.30E-03 | K | 1.68E-02 |
| Fe | 83.928 | SiO_2_ | 39.233 | SiO_2_ | 6.28E-01 | Na | 8.88E-03 |
| Mn | 8.3114 | NaAlO_2_ | 0.91683 | NaAlO_2_ | 9.68E-05 | Others | 0.000873 |
| S | 0.48642 | KAlO_2_ | 1.3309 | KAlO_2_ | 1.22E-04 |  |  |
| Si | 1.9313 | CaO | 40.168 | CaO | 5.5049 |  |  |
| V | 0.23872 | MgO | 6.636 | FeO | 0.12283 |  |  |
| Others | 0.17 | MnO | 1.9298 | MgO | 0.10733 |  |  |
|  |  | Ti_2_O_3_ | 0.25038 | MnO | 5.7488 |  |  |
|  |  | TiO2 | 0.14027 | Cu_2_O | 0.48012 |  |  |
|  |  | Al_2_S_3_ | 0.1287 | Na_2_S | 1.0442 |  |  |
|  |  | SiS_2_ | 0.99991 | K_2_S | 1.0231 |  |  |
|  |  | CaS | 0.85815 | SiS_2_ | 4.9526 |  |  |
|  |  | MgS | 0.15413 | CaS | 36.639 |  |  |
|  |  | Others | 0.23366 | MgS | 0.77658 |  |  |
|  |  |  |  | MnS | 36.477 |  |  |
|  |  |  |  | Cu2S | 0.48012 |  |  |
|  |  |  |  | Mn_2_S_3_ | 0.26188 |  |  |
|  |  |  |  | K_2_MgSiO_4_ | 0.61504 |  |  |
|  |  |  |  | K_2_MgSiS_4_ | 4.2328 |  |  |
|  |  |  |  | Others | 0.569963 |  |  |

S-Table 2 FactSage expected distribution of ID 2 composition of metal phase, slag phase, and gas phase.

| ID 2 | | | | | | | |
| --- | --- | --- | --- | --- | --- | --- | --- |
| Metal (41.733 g) | | Slag 1 (36.881 g) | | Slag 2 (1.0741 g) | | Gas (1 atm) | |
| Phase | wt. % | Phase | wt. % | Phase | wt. % | Phase | Mass (mol) |
| Al | 4.96E-04 | Na_2_O | 0.72508 | Na_2_O | 0.14259 | CO | 0.94506 |
| C | 4.5976 | K_2_O | 0.86106 | K_2_O | 1.58E-01 | Zn | 3.07E-02 |
| Cr | 0.1162 | Al_2_O_3_ | 5.2855 | Al_2_O_3_ | 5.86E-03 | K | 1.55E-02 |
| Cu | 0.46094 | SiO_2_ | 39.177 | SiO_2_ | 6.16E-01 | Na | 7.80E-03 |
| Fe | 84.1 | NaAlO_2_ | 0.76497 | NaAlO_2_ | 8.45E-05 | Others | 0.0009052 |
| Mn | 7.7653 | KAlO_2_ | 1.1547 | KAlO_2_ | 1.11E-04 |  |  |
| S | 0.48369 | CaO | 40.021 | CaO | 5.6049 |  |  |
| Si | 1.8398 | MgO | 7.3815 | FeO | 0.12724 |  |  |
| V | 0.48569 | MnO | 1.7865 | MgO | 0.12287 |  |  |
| Others | 0.15 | Ti_2_O_3_ | 0.37626 | MnO | 5.5187 |  |  |
|  |  | TiO2 | 0.20069 | Cu2O | 0.10486 |  |  |
|  |  | Al_2_S_3_ | 0.12231 | Na_2_S | 0.93987 |  |  |
|  |  | SiS_2_ | 0.94481 | K_2_S | 0.96516 |  |  |
|  |  | CaS | 0.80902 | SiS_2_ | 4.9526 |  |  |
|  |  | MgS | 0.16222 | CaS | 37.743 |  |  |
|  |  | Others | 0.22738 | MgS | 0.8995 |  |  |
|  |  |  |  | MnS | 35.43 |  |  |
|  |  |  |  | Cu2S | 0.61048 |  |  |
|  |  |  |  | Mn_2_S_3_ | 0.2597 |  |  |
|  |  |  |  | K_2_MgSiO_4_ | 0.60395 |  |  |
|  |  |  |  | K_2_MgSiS_4_ | 4.2054 |  |  |
|  |  |  |  | Others | 0.989128 |  |  |

S-Table 3 FactSage expected distribution of ID 3 composition of metal phase, slag phase, and gas phase.

| ID 3 | | | | | | | |
| --- | --- | --- | --- | --- | --- | --- | --- |
| Metal (40.741 g) | | Slag 1 (39.637 g) | | Slag 2 (0.63217 g) | | Gas (1 atm) | |
| Phase | wt. % | Phase | wt. % | Phase | wt. % | Phase | Mass (mol) |
| Al | 4.84E-04 | Na_2_O | 0.63971 | CaS | 100 | CO | 0.94961 |
| C | 4.6726 | K_2_O | 0.7963 |  |  | Zn | 2.77E-02 |
| Cr | 0.16538 | Al_2_O_3_ | 5.2813 |  |  | K | 1.47E-02 |
| Cu | 0.54328 | SiO_2_ | 39.022 |  |  | Na | 7.06E-03 |
| Fe | 83.811 | NaAlO_2_ | 0.63294 |  |  | Others | 0.0009414 |
| Mn | 7.6906 | KAlO_2_ | 0.98998 |  |  |  |  |
| P | 0.13102 | CaO | 39.955 |  |  |  |  |
| S | 0.48783 | MgO | 8.1133 |  |  |  |  |
| Si | 1.6923 | MnO | 1.7154 |  |  |  |  |
| V | 0.74322 | Ti_2_O_3_ | 0.48584 |  |  |  |  |
| Others | 0.06 | TiO2 | 0.25078 |  |  |  |  |
|  |  | Al_2_S_3_ | 0.11327 |  |  |  |  |
|  |  | SiS_2_ | 0.87221 |  |  |  |  |
|  |  | CaS | 0.74859 |  |  |  |  |
|  |  | MgS | 0.16526 |  |  |  |  |
|  |  | Others | 0.21812 |  |  |  |  |

S-Table 4 FactSage expected distribution of ID 4 composition of metal phase, slag phase, and gas phase.

| ID 4 | | | | | | | |
| --- | --- | --- | --- | --- | --- | --- | --- |
| Metal (39.511 g) | | Slag 1 (43.007 g) | | Slag 2 (0.25442 g) | | Gas (1 atm) | |
| Phase | wt. % | Phase | wt. % | Phase | wt. % | Phase | Mass (mol) |
| Al | 4.61E-04 | Na_2_O | 0.54211 | CaS | 100 | CO | 0.95862 |
| C | 4.5983 | K_2_O | 0.7519 |  |  | Zn | 2.31E-02 |
| Cr | 0.22354 | Al_2_O_3_ | 5.4156 |  |  | K | 1.20E-02 |
| Cu | 0.69514 | SiO_2_ | 39.733 |  |  | Na | 5.29E-03 |
| Fe | 84.435 | NaAlO_2_ | 0.55366 |  |  | Others | 0.0009864 |
| Mn | 6.411 | KAlO_2_ | 0.9641 |  |  |  |  |
| P | 0.1763 | CaO | 39.036 |  |  |  |  |
| S | 0.47805 | MgO | 8.567 |  |  |  |  |
| Si | 1.8917 | MnO | 1.5427 |  |  |  |  |
| V | 1.0193 | Ti_2_O_3_ | 0.59393 |  |  |  |  |
| Others | 0.07 | TiO2 | 0.29803 |  |  |  |  |
|  |  | Al_2_S_3_ | 0.10808 |  |  |  |  |
|  |  | SiS_2_ | 0.82642 |  |  |  |  |
|  |  | CaS | 0.68057 |  |  |  |  |
|  |  | MgS | 0.16238 |  |  |  |  |
|  |  | Others | 0.22452 |  |  |  |  |

S-Table 5 FactSage expected distribution of ID 5 composition of metal phase, slag phase, and gas phase.

| ID 5 | | | | | |
| --- | --- | --- | --- | --- | --- |
| Metal (38.506 g) | | Slag (45.720 g) | | Gas (1 atm) | |
| Phase | wt. % | Phase | wt. % | Phase | Mass (mol) |
| Al | 4.44E-04 | Na_2_O | 0.45848 | CO | 0.96611 |
| C | 4.5314 | K_2_O | 0.70226 | Zn | 1.90E-02 |
| Cr | 0.27487 | Al_2_O_3_ | 5.557 | K | 9.88E-03 |
| Cu | 0.83249 | SiO_2_ | 40.423 | Na | 4.02E-03 |
| Fe | 84.961 | NaAlO_2_ | 0.48401 | Others | 0.0010355 |
| Mn | 5.3119 | KAlO_2_ | 0.93084 |  |  |
| P | 0.21638 | CaO | 38.326 |  |  |
| S | 0.45513 | MgO | 8.9178 |  |  |
| Si | 2.0769 | MnO | 1.359 |  |  |
| V | 1.2613 | Ti_2_O_3_ | 0.67722 |  |  |
| Others | 0.08 | TiO2 | 0.33143 |  |  |
|  |  | Al_2_S_3_ | 9.98E-02 |  |  |
|  |  | SiS_2_ | 0.75643 |  |  |
|  |  | CaS | 0.60117 |  |  |
|  |  | MgS | 0.15207 |  |  |
|  |  | Others | 0.223507 |  |  |

S-Table 6 FactSage expected distribution of ID 6 composition of metal phase, slag phase, and gas phase.

| ID 6 | | | | | | | |
| --- | --- | --- | --- | --- | --- | --- | --- |
| Metal (37.972 g) | | Slag 1 (44.903 g) | | Slag 2 (0.16455 g) | | Gas (1 atm) | |
| Phase | wt. % | Phase | wt. % | Phase | wt. % | Phase | Mass (mol) |
| Al | 4.75E-04 | Na_2_O | 0.46248 | CaS | 100 | CO | 0.96161 |
| C | 4.7152 | K_2_O | 0.65272 |  |  | Zn | 2.07E-02 |
| Cr | 0.27175 | Al_2_O_3_ | 5.3772 |  |  | K | 1.17E-02 |
| Cu | 0.74355 | SiO_2_ | 39.203 |  |  | Na | 5.01E-03 |
| Fe | 84.631 | NaAlO_2_ | 0.44296 |  |  | Others | 0.0009889 |
| Mn | 6.0405 | KAlO_2_ | 0.77402 |  |  |  |  |
| P | 0.21195 | CaO | 39.878 |  |  |  |  |
| S | 0.42455 | MgO | 9.0659 |  |  |  |  |
| Si | 1.6092 | MnO | 1.3449 |  |  |  |  |
| V | 1.2788 | Ti_2_O_3_ | 0.66926 |  |  |  |  |
| Others | 0.07 | TiO2 | 0.32599 |  |  |  |  |
|  |  | Al_2_S_3_ | 9.59E-02 |  |  |  |  |
|  |  | SiS_2_ | 0.72898 |  |  |  |  |
|  |  | CaS | 0.62158 |  |  |  |  |
|  |  | MgS | 0.15362 |  |  |  |  |
|  |  | Others | 0.203443 |  |  |  |  |

S-Table 7 FactSage expected distribution of ID 7 composition of metal phase, slag phase, and gas phase.

| ID 7 | | | | | |
| --- | --- | --- | --- | --- | --- |
| Metal (37.253 g) | | Slag 1 (48.410 g) | | Gas (1 atm) | |
| Phase | wt. % | Phase | wt. % | Phase | Mass (mol) |
| Al | 4.41E-04 | Na_2_O | 0.37024 | CO | 0.97205 |
| C | 4.5688 | K_2_O | 0.61406 | Zn | 1.49E-02 |
| Cr | 0.33599 | Al_2_O_3_ | 5.6415 | K | 8.70E-03 |
| Cu | 0.94496 | SiO_2_ | 40.65 | Na | 3.27E-03 |
| Fe | 85.367 | NaAlO_2_ | 0.38482 | Others | 0.0010527 |
| Mn | 4.4871 | KAlO_2_ | 0.79455 |  |  |
| P | 0.26278 | CaO | 38.478 |  |  |
| S | 0.37396 | MgO | 9.4298 |  |  |
| Si | 2.0052 | MnO | 1.1194 |  |  |
| V | 1.5704 | Ti_2_O_3_ | 0.76776 |  |  |
| Others | 0.08 | TiO2 | 0.36035 |  |  |
|  |  | Al_2_S_3_ | 7.43E-02 |  |  |
|  |  | SiS_2_ | 0.55817 |  |  |
|  |  | CaS | 0.44287 |  |  |
|  |  | MgS | 0.11799 |  |  |
|  |  | Others | 0.196159 |  |  |

S-Table 8 FactSage expected distribution of ID 8 composition of metal phase, slag phase, and gas phase.

| ID 8 | | | | | |
| --- | --- | --- | --- | --- | --- |
| Metal (37.176 g) | | Slag 1 (48.158 g) | | Gas (1 atm) | |
| Phase | wt. % | Phase | wt. % | Phase | Mass (mol) |
| Al | 4.67E-04 | Na_2_O | 0.3795 | CO | 0.9683 |
| C | 4.72 | K_2_O | 0.58417 | Zn | 1.66E-02 |
| Cr | 0.331 | Al_2_O_3_ | 5.4578 | K | 1.01E-02 |
| Cu | 0.86293 | SiO_2_ | 39.489 | Na | 3.99E-03 |
| Fe | 85.074 | NaAlO_2_ | 0.36356 | Others | 0.0010163 |
| Mn | 5.1069 | KAlO_2_ | 0.68934 |  |  |
| P | 0.25723 | CaO | 39.733 |  |  |
| S | 0.37946 | MgO | 9.4862 |  |  |
| Si | 1.6158 | MnO | 1.147 |  |  |
| V | 1.5735 | Ti_2_O_3_ | 0.75258 |  |  |
| Others | 0.08 | TiO2 | 0.35682 |  |  |
|  |  | Al_2_S_3_ | 8.26E-02 |  |  |
|  |  | SiS_2_ | 0.62285 |  |  |
|  |  | CaS | 0.52533 |  |  |
|  |  | MgS | 0.13635 |  |  |
|  |  | Others | 0.193895 |  |  |

S-Table 9 FactSage expected distribution of ID 9 composition of metal phase, slag phase, and gas phase.

| ID 9 | | | | | |
| --- | --- | --- | --- | --- | --- |
| Metal (35.530 g) | | Slag 1 (50.641 g) | | Gas (1 atm) | |
| Phase | wt. % | Phase | wt. % | Phase | Mass (mol) |
| Al | 4.48E-04 | Na_2_O | 0.28741 | CO | 0.97712 |
| C | 4.6754 | K_2_O | 0.51407 | Zn | 1.11E-02 |
| Cr | 0.40369 | Al_2_O_3_ | 5.6417 | K | 8.00E-03 |
| Cu | 1.036 | SiO_2_ | 40.361 | Na | 2.76E-03 |
| Fe | 85.673 | NaAlO_2_ | 0.28449 | Others | 0.0010553 |
| Mn | 3.8353 | KAlO_2_ | 0.62556 |  |  |
| P | 0.31337 | CaO | 39.17 |  |  |
| S | 0.28791 | MgO | 9.9495 |  |  |
| Si | 1.7626 | MnO | 0.89422 |  |  |
| V | 1.9252 | Ti_2_O_3_ | 0.84962 |  |  |
| Others | 0.09 | TiO2 | 0.38655 |  |  |
|  |  | Al_2_S_3_ | 5.31E-02 |  |  |
|  |  | SiS_2_ | 0.39597 |  |  |
|  |  | CaS | 0.32211 |  |  |
|  |  | MgS | 8.90E-02 |  |  |
|  |  | Others | 0.175741 |  |  |

S-Table 10 FactSage expected distribution of ID 10 composition of metal phase, slag phase, and gas phase.

| ID 10 | | | | | |
| --- | --- | --- | --- | --- | --- |
| Metal (34.700 g) | | Slag 1 (53.474 g) | | Gas (1 atm) | |
| Phase | wt. % | Phase | wt. % | Phase | Mass (mol) |
| Al | 4.59E-04 | Na_2_O | 0.22286 | CO | 0.98136 |
| C | 4.7893 | K_2_O | 0.43098 | Zn | 7.74E-03 |
| Cr | 0.46601 | Al_2_O_3_ | 5.6076 | K | 7.50E-03 |
| Cu | 1.1066 | SiO_2_ | 39.928 | Na | 2.36E-03 |
| Fe | 85.877 | NaAlO_2_ | 0.20818 | Others | 0.0010496 |
| Mn | 3.3334 | KAlO_2_ | 0.48894 |  |  |
| P | 0.35959 | CaO | 39.925 |  |  |
| S | 0.21494 | MgO | 10.375 |  |  |
| Si | 1.5082 | MnO | 0.71915 |  |  |
| V | 2.2559 | Ti_2_O_3_ | 0.90843 |  |  |
| Others | 0.09 | TiO2 | 0.41201 |  |  |
|  |  | Al_2_S_3_ | 3.73E-02 |  |  |
|  |  | SiS_2_ | 0.27689 |  |  |
|  |  | CaS | 0.23208 |  |  |
|  |  | MgS | 6.56E-02 |  |  |
|  |  | Others | 0.162 |  |  |


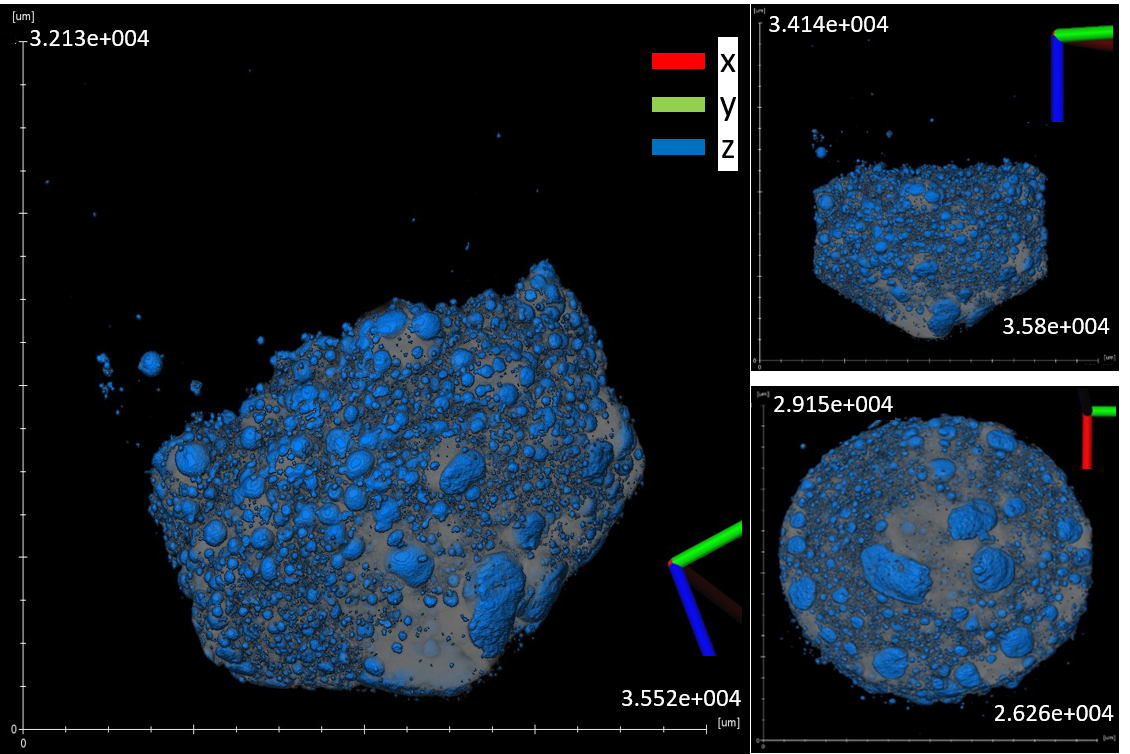


S-Figure 5 CT images of smelting products from the tube furnace experiment of mixture ID 1: Gray regions indicate slag, while blue regions represent the metal phases


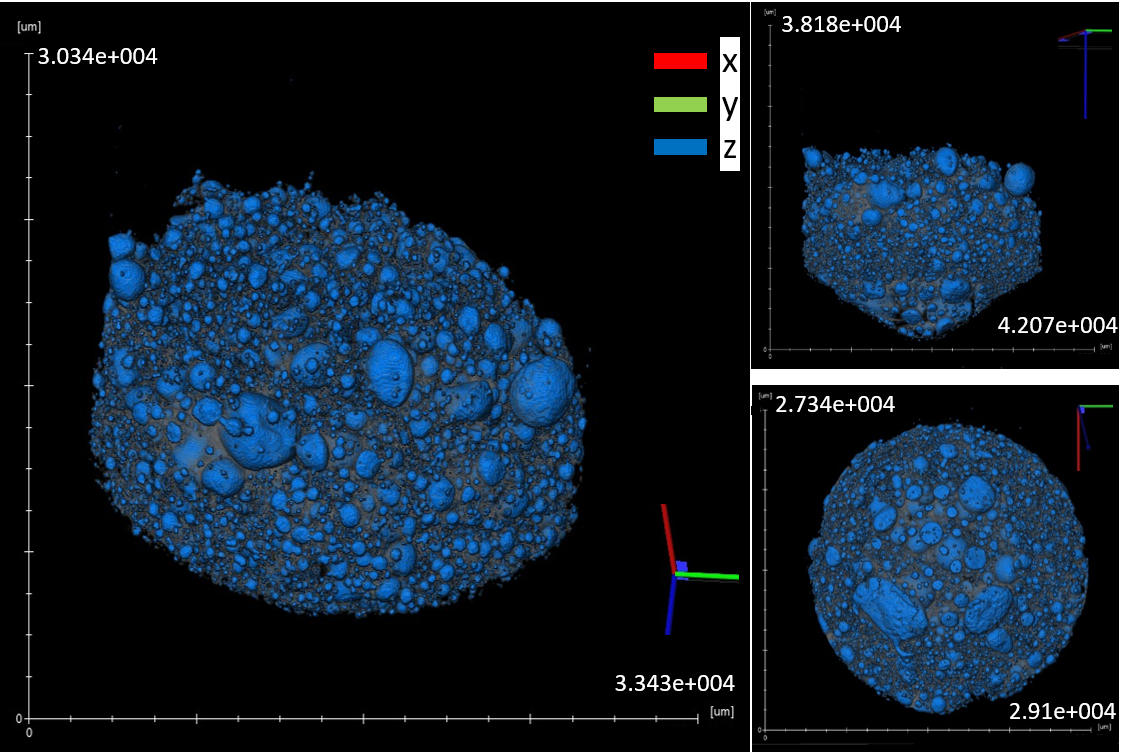


S-Figure 6 CT images of smelting products from the tube furnace experiment of mixture ID 2: Gray regions indicate slag, while blue regions represent the metal phase


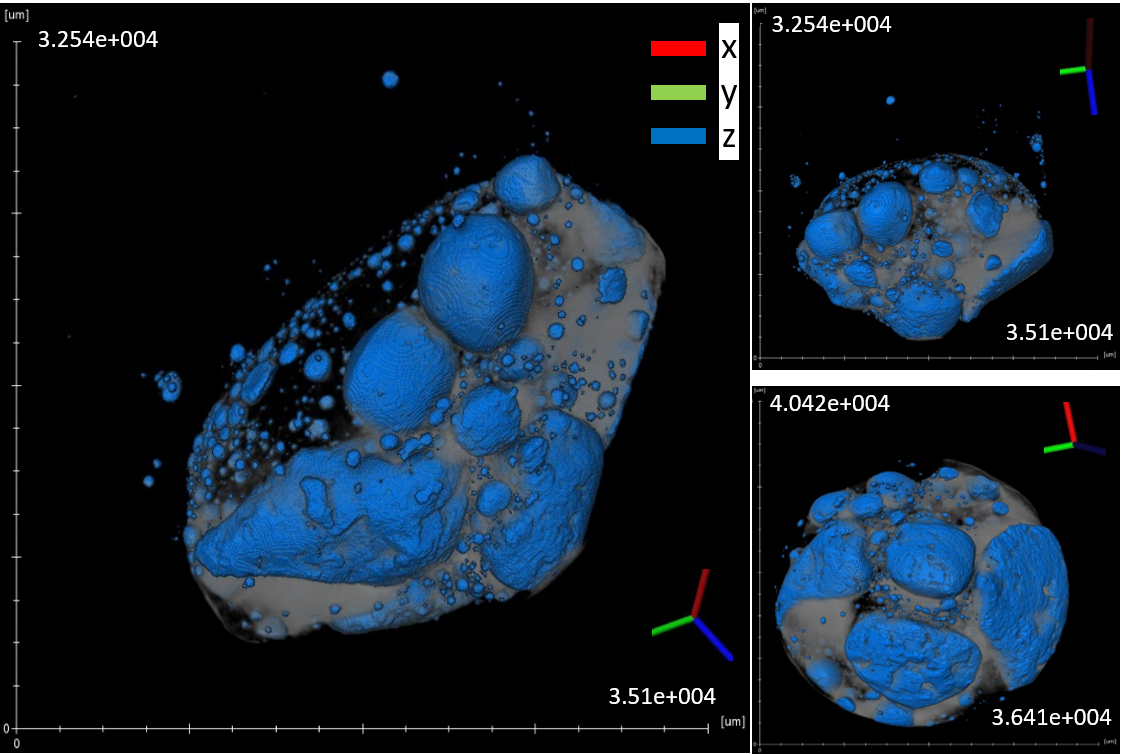


S-Figure 7 CT images of smelting products from the tube furnace experiment of mixture ID 3: Gray regions indicate slag, while blue regions represent the metal phase


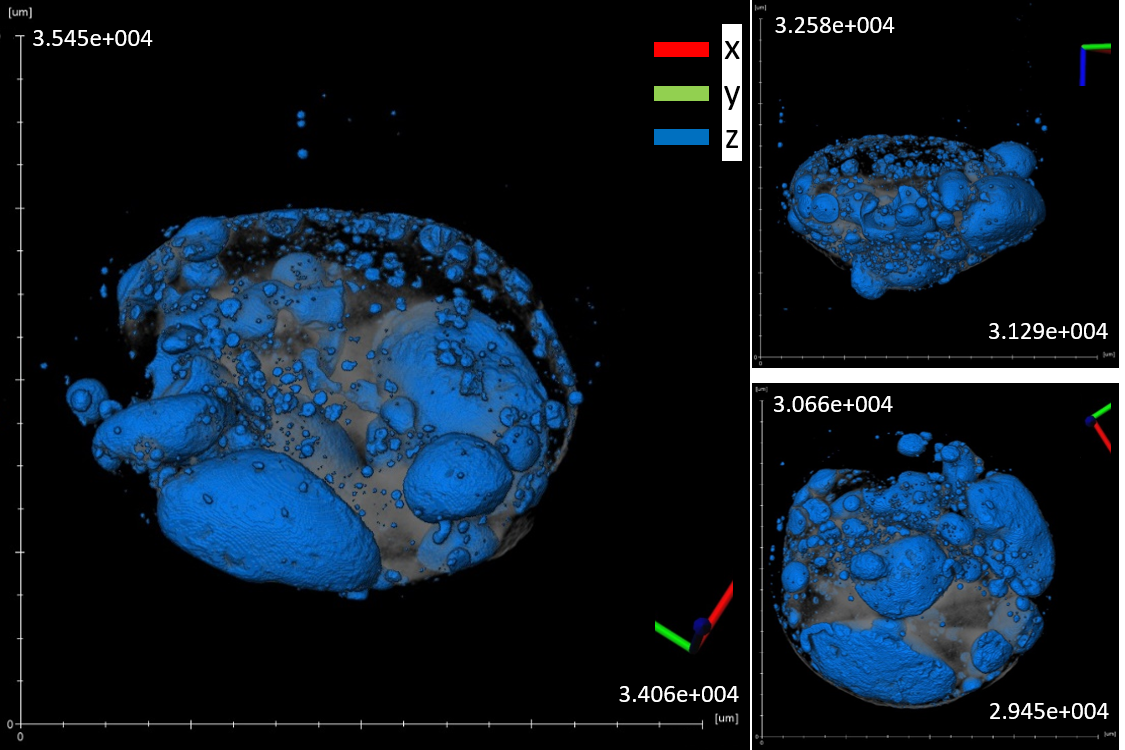


S-Figure 8 CT images of smelting products from the tube furnace experiment of mixture ID 4: Gray regions indicate slag, while blue regions represent the metal phase


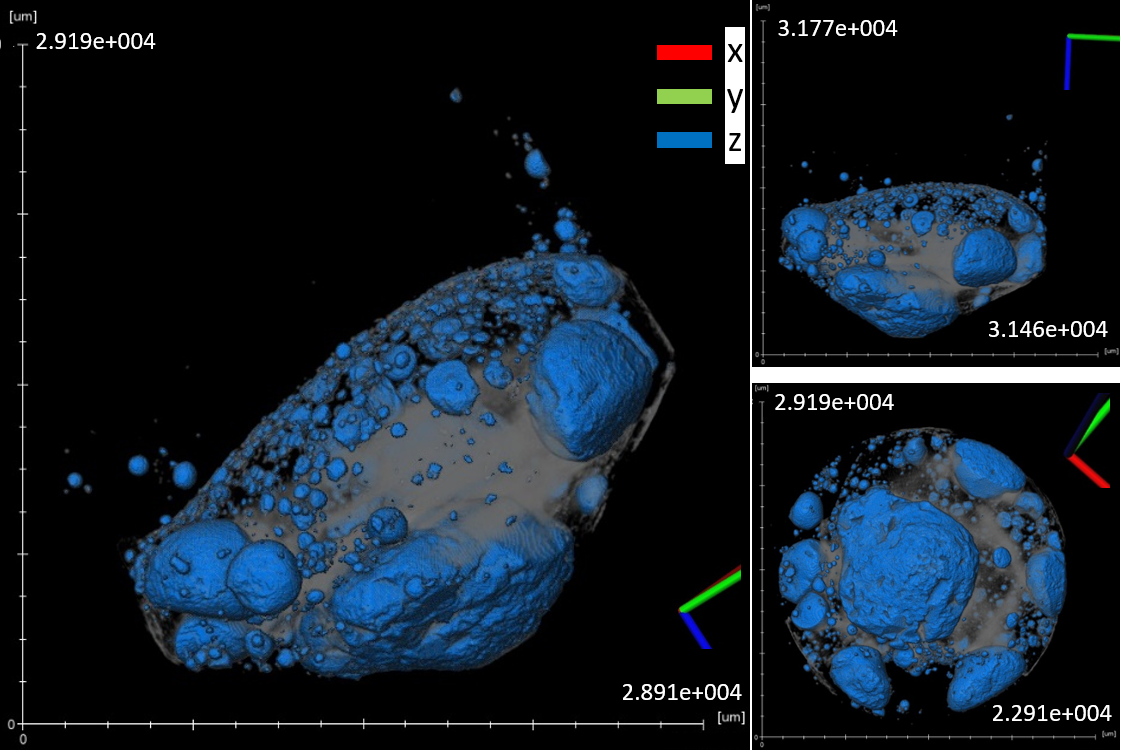


S-Figure 9 CT images of smelting products from the tube furnace experiment of mixture ID 5: Gray regions indicate slag, while blue regions represent the metal phase


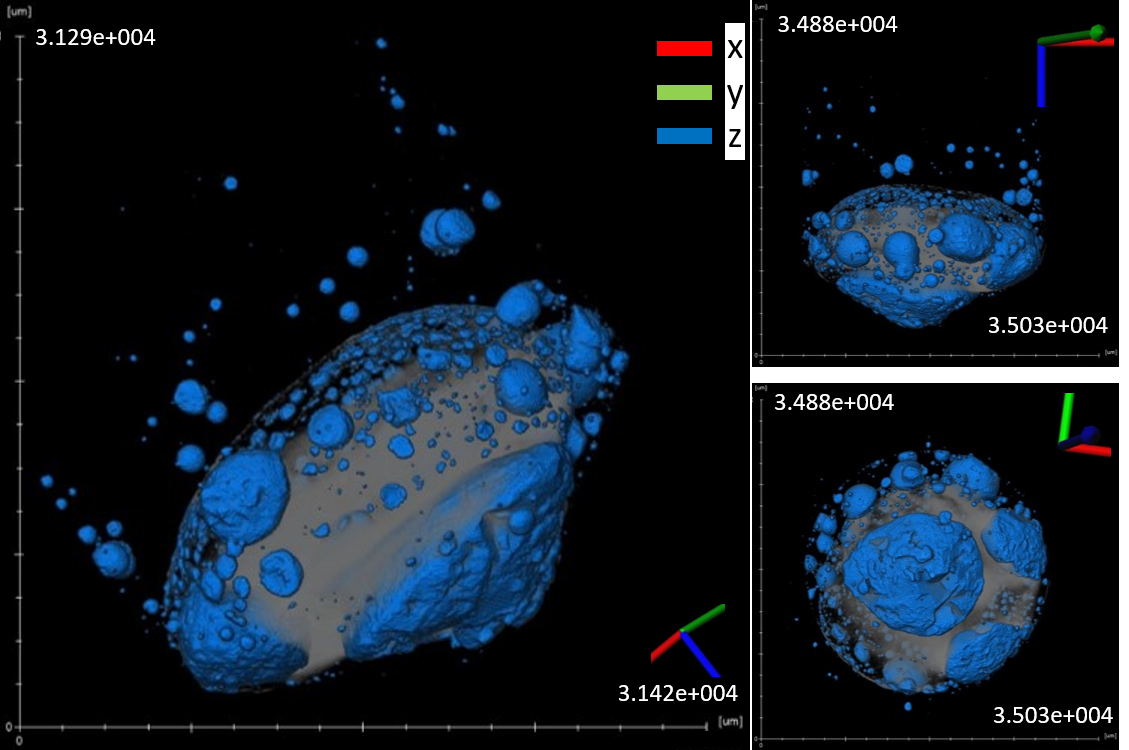


S-Figure 10 CT images of smelting products from the tube furnace experiment of mixture ID 6: Gray regions indicate slag, while blue regions represent the metal phase


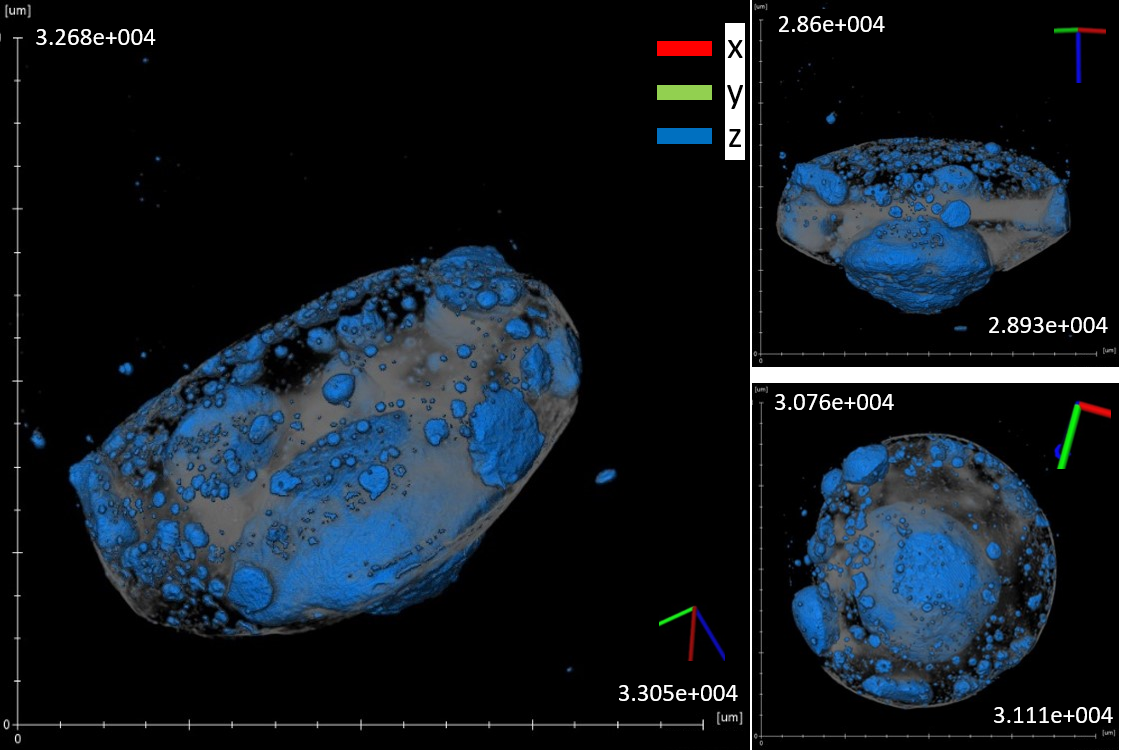


S-Figure 11 CT images of smelting products from the tube furnace experiment of mixture ID 7: Gray regions indicate slag, while blue regions represent the metal phase


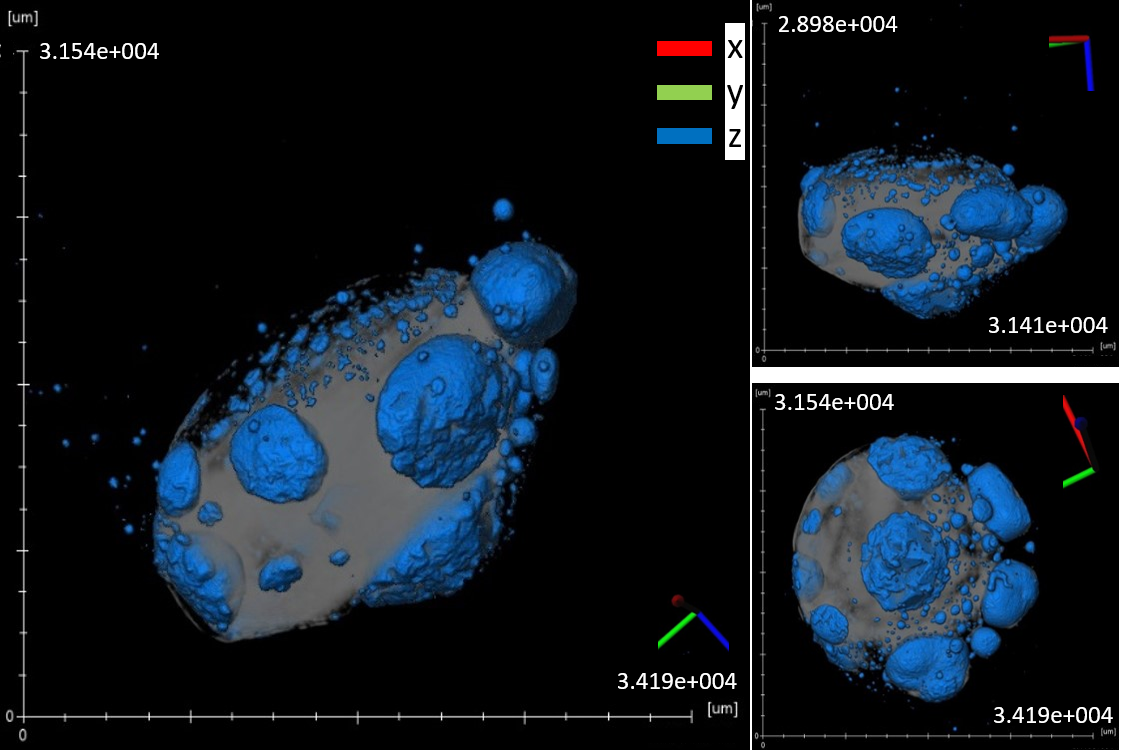


S-Figure 12 CT images of smelting products from the tube furnace experiment of mixture ID 8: Gray regions indicate slag, while blue regions represent the metal phase


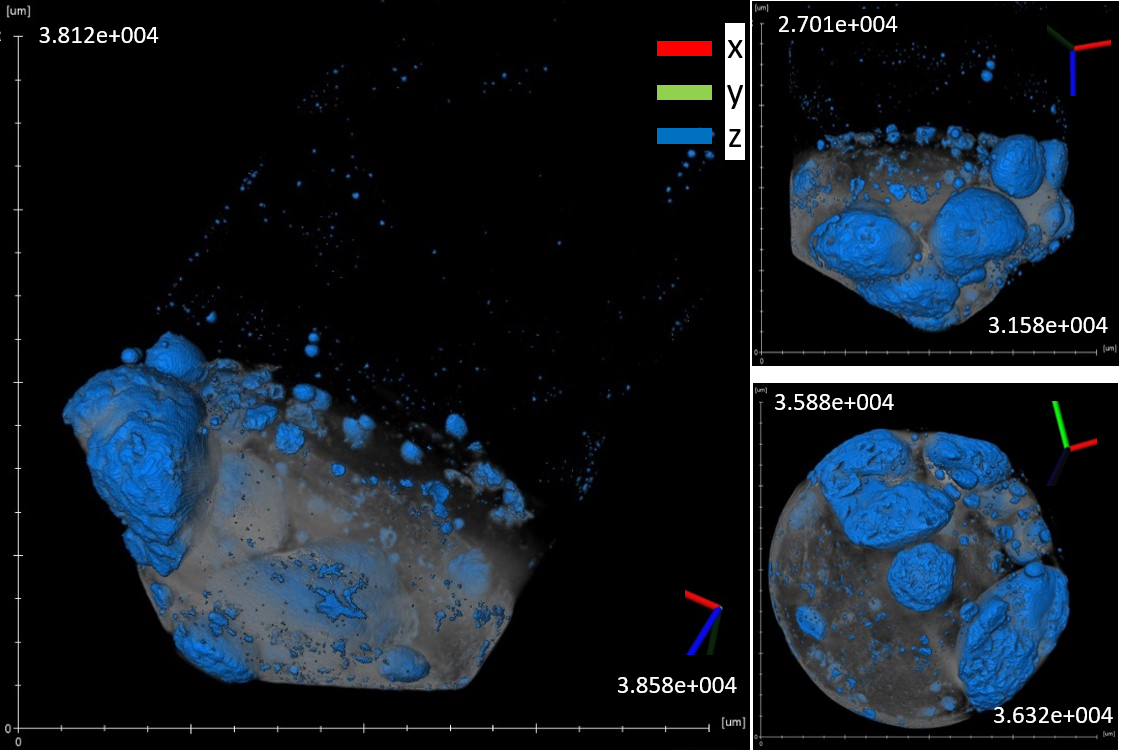


S-Figure 13 CT images of smelting products from the tube furnace experiment of mixture ID 10: Gray regions indicate slag, while blue regions represent the metal phase

S-Table 11 Elemental composition (wt %) of Metal Phase

| **Elements** | **ID** | | | | | | | | | |
| --- | --- | --- | --- | --- | --- | --- | --- | --- | --- | --- |
|  | **1** | **2** | **3** | **4** | **5** | **6** | **7** | **8** | **9** | **10** |
| Al | 0.04 | 0.02 | 0.03 | 0.02 | 0.02 | 0.03 | 0.02 | 0.02 | 0.02 | 0.02 |
| Ca | 0.06 | 0.00 | 0.01 | 0.00 | 0.01 | 0.01 | 0.01 | 0.00 | 0.01 | 0.01 |
| Cu | 0.64 | 0.63 | 0.72 | 0.78 | 0.96 | 0.88 | 1.04 | 0.95 | 1.16 | 1.20 |
| Fe | 89.09 | 88.06 | 87.61 | 88.99 | 86.73 | 87.83 | 88.13 | 91.08 | 91.41 | 91.75 |
| K | 0.00 | 0.00 | 0.00 | 0.00 | 0.00 | 0.00 | 0.00 | 0.00 | 0.00 | 0.00 |
| Mg | 0.01 | 0.00 | 0.00 | 0.00 | 0.00 | 0.00 | 0.00 | 0.00 | 0.00 | 0.00 |
| Mn | 5.63 | 7.15 | 5.67 | 4.75 | 5.35 | 4.79 | 4.01 | 3.38 | 3.10 | 3.02 |
| Na | 0.00 | 0.00 | 0.01 | 0.01 | 0.01 | 0.01 | 0.01 | 0.01 | 0.00 | 0.01 |
| P | 0.15 | 0.21 | 0.13 | 0.13 | 0.14 | 0.12 | 0.11 | 0.15 | 0.12 | 0.16 |
| S | 0.10 | 0.11 | 0.08 | 0.10 | 0.12 | 0.12 | 0.15 | 0.15 | 0.18 | 0.18 |
| Si | 3.12 | 2.50 | 2.66 | 2.70 | 3.87 | 2.85 | 3.40 | 1.71 | 2.26 | 2.13 |
| Ti | 0.00 | 0.22 | 0.14 | 0.18 | 0.19 | 0.20 | 0.25 | 0.16 | 0.18 | 0.12 |
| V | 0.33 | 0.99 | 0.69 | 1.07 | 1.41 | 1.02 | 1.26 | 1.41 | 1.20 | 1.24 |

S-Table 12 Elemental composition (wt %) of Slag Phase

| **Elements** | **ID** | | | | | | | | | |
| --- | --- | --- | --- | --- | --- | --- | --- | --- | --- | --- |
|  | **1** | **2** | **3** | **4** | **5** | **6** | **7** | **8** | **9** | **10** |
| Al | 2.65 | 2.79 | 2.84 | 2.94 | 2.90 | 2.76 | 2.82 | 2.77 | 2.80 | 2.77 |
| Ca | 26.60 | 28.78 | 27.46 | 26.50 | 27.13 | 26.88 | 27.10 | 26.60 | 27.50 | 28.54 |
| Cu | 0.01 | 0.00 | 0.01 | 0.01 | 0.01 | 0.01 | 0.00 | 0.00 | 0.01 | 0.00 |
| Fe | 0.03 | 0.02 | 0.05 | 0.02 | 0.02 | 0.03 | 0.02 | 0.02 | 0.03 | 0.04 |
| K | 0.00 | 0.00 | 0.00 | 0.00 | 0.00 | 0.00 | 0.00 | 0.00 | 0.00 | 0.00 |
| Mg | 7.15 | 6.96 | 6.54 | 6.31 | 6.47 | 6.38 | 6.33 | 6.15 | 6.45 | 6.49 |
| Mn | 1.87 | 0.62 | 1.00 | 1.04 | 0.74 | 1.13 | 0.61 | 0.94 | 0.56 | 0.33 |
| Na | 0.00 | 0.00 | 0.00 | 0.00 | 0.00 | 0.00 | 0.00 | 0.00 | 0.00 | 0.00 |
| P | 0.01 | 0.01 | 0.01 | 0.00 | 0.00 | 0.00 | 0.00 | 0.00 | 0.00 | 0.00 |
| S | 3.38 | 2.87 | 2.95 | 2.23 | 1.72 | 1.89 | 1.30 | 1.35 | 1.02 | 0.80 |
| Si | 15.31 | 14.73 | 15.60 | 16.33 | 16.78 | 16.86 | 16.90 | 17.07 | 17.30 | 17.37 |
| Ti | 0.31 | 0.28 | 0.31 | 0.35 | 0.34 | 0.44 | 0.33 | 0.48 | 0.37 | 0.33 |
| V | 0.01 | 0.01 | 0.01 | 0.01 | 0.01 | 0.01 | 0.02 | 0.01 | 0.02 | 0.02 |

S-Table 13 Recovery (%) of Elements in Metal Phase

| **Elements** | **ID** | | | | | | | | | |
| --- | --- | --- | --- | --- | --- | --- | --- | --- | --- | --- |
|  | **1** | **2** | **3** | **4** | **5** | **6** | **7** | **8** | **9** | **10** |
| Al | 1.52 | 0.58 | 0.85 | 0.54 | 0.46 | 0.78 | 0.43 | 0.46 | 0.41 | 0.40 |
| Ca | 0.27 | 0.00 | 0.03 | 0.00 | 0.03 | 0.03 | 0.03 | 0.00 | 0.02 | 0.02 |
| Cu | 159.81 | 108.58 | 112.39 | 103.62 | 99.13 | 110.14 | 96.24 | 98.39 | 100.62 | 99.30 |
| Fe | 112.20 | 92.01 | 95.39 | 104.44 | 93.60 | 103.98 | 96.38 | 102.73 | 102.45 | 104.31 |
| K | 0.00 | 0.00 | 0.00 | 0.00 | 0.00 | 0.00 | 0.00 | 0.00 | 0.00 | 0.00 |
| Mg | 0.33 | 0.00 | 0.00 | 0.00 | 0.00 | 0.00 | 0.00 | 0.00 | 0.00 | 0.00 |
| Mn | 56.83 | 64.45 | 57.22 | 60.48 | 73.68 | 65.64 | 65.90 | 51.49 | 61.14 | 69.60 |
| Na | 0.00 | 0.00 | 0.91 | 1.12 | 1.20 | 1.24 | 1.43 | 1.39 | 0.00 | 2.18 |
| P | 279.42 | 200.24 | 92.66 | 74.88 | 59.20 | 56.94 | 39.12 | 55.95 | 37.25 | 43.71 |
| S | 3.80 | 3.80 | 3.16 | 5.06 | 6.75 | 6.83 | 10.59 | 9.95 | 16.59 | 22.31 |
| Si | 19.74 | 12.02 | 12.36 | 12.01 | 14.38 | 12.14 | 11.83 | 6.38 | 7.56 | 6.86 |
| Ti | 0.00 | 54.53 | 25.95 | 26.96 | 20.90 | 24.69 | 23.13 | 15.52 | 14.54 | 8.53 |
| V | 149.19 | 181.98 | 85.94 | 105.36 | 99.66 | 78.68 | 73.19 | 84.06 | 59.42 | 52.74 |

S-Table 14 Recovery (%) of Elements in Slag Phase

| **Elements** | **ID** | | | | | | | | | |
| --- | --- | --- | --- | --- | --- | --- | --- | --- | --- | --- |
|  | **1** | **2** | **3** | **4** | **5** | **6** | **7** | **8** | **9** | **10** |
| Al | 64.92 | 85.43 | 73.79 | 77.45 | 69.95 | 77.51 | 71.74 | 73.53 | 76.36 | 79.43 |
| Ca | 76.35 | 101.73 | 80.09 | 82.56 | 78.96 | 84.11 | 82.59 | 78.94 | 87.17 | 90.43 |
| Cu | 1.61 | 0.00 | 1.44 | 1.29 | 1.09 | 1.35 | 0.00 | 0.00 | 1.15 | 0.00 |
| Fe | 0.02 | 0.02 | 0.05 | 0.02 | 0.02 | 0.04 | 0.03 | 0.03 | 0.04 | 0.07 |
| K | 0.00 | 0.00 | 0.00 | 0.00 | 0.00 | 0.00 | 0.00 | 0.00 | 0.00 | 0.00 |
| Mg | 152.27 | 162.73 | 115.84 | 108.59 | 96.85 | 105.53 | 94.20 | 91.27 | 96.28 | 94.61 |
| Mn | 12.16 | 5.89 | 9.29 | 12.84 | 10.78 | 16.66 | 11.90 | 16.57 | 14.58 | 10.93 |
| Na | 0.00 | 0.00 | 0.00 | 0.00 | 0.00 | 0.00 | 0.00 | 0.00 | 0.00 | 0.00 |
| P | 12.00 | 10.04 | 6.56 | 0.00 | 0.00 | 0.00 | 0.00 | 0.00 | 0.00 | 0.00 |
| S | 82.84 | 104.36 | 107.40 | 109.40 | 102.44 | 115.71 | 108.94 | 103.64 | 124.15 | 142.48 |
| Si | 62.41 | 74.63 | 66.74 | 70.43 | 65.97 | 77.26 | 69.82 | 73.66 | 76.39 | 80.44 |
| Ti | 96.63 | 73.10 | 52.91 | 50.83 | 39.57 | 58.41 | 36.24 | 53.90 | 39.45 | 33.71 |
| V | 2.91 | 1.94 | 1.15 | 0.95 | 0.75 | 0.83 | 1.38 | 0.69 | 1.31 | 1.22 |


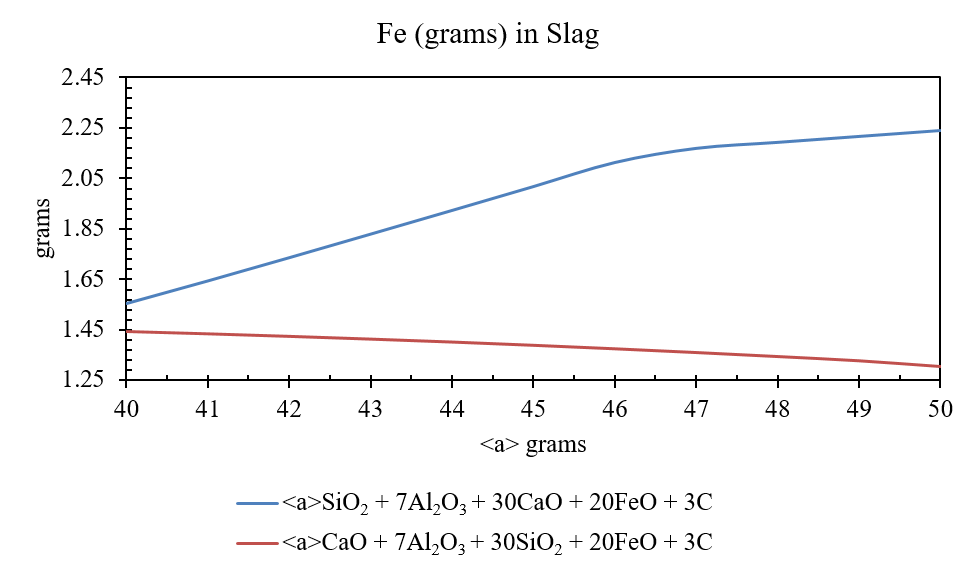


S-Figure 14 Amount of Fe left in the slag after reaction at 1450°C, using FactSage Equilibrium Module with FToxid, FactPS, FTstel, and FTmisc databases


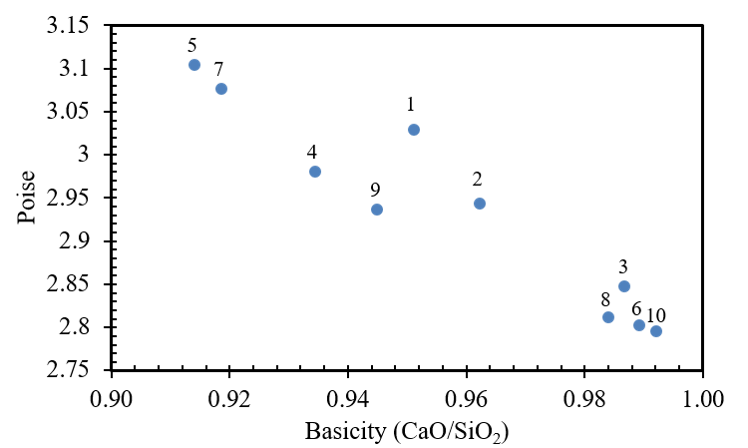


S-Figure 15 Basicity vs. Viscosity of all mixtures at 1450°C

S-Table 15 Comparison table of metal nugget recovered with some standards

| Type | C | Si | Mn | S | P | Al | Ni | V | Cu | Country/Reference |
| --- | --- | --- | --- | --- | --- | --- | --- | --- | --- | --- |
| Metal nugget | 1.65 | 6.39 | 2.28 | 0.44 | - | - | - | 1.81 | 1.09 | 2-kg smelting experiment |
| FeSi10 | ≤2 | 8.0-13.0 | ≤3 | ≤0.06 | ≤0.15 | ≤0.2 | - | - | - | Database of Steel and Alloy (Marochnik)  Accessed on 13^th^ November 2025: <https://www.splav-kharkov.com/en/e_mat_start.php?name_id=2021>  Grudinsky, P., Yurtaeva, A., Pankratov, D., Pasechnik, L., Musaelyan, R., & Dyubanov, V. (2024). The Waelz Slag from Electric Arc Furnace Dust Processing: Characterization and Magnetic Separation Studies. Materials, 17(10), 2224. <https://doi.org/10.3390/ma17102224> |
| Cast Iron CHG8D3 (GOST 7769-82) | 3-3.8 | 2-2.5 | 7-9 | ≤0.1 | ≤0.3 | 0.5-1 | 0.8-1.5 | - | 2.5-3.5 | Evek GmbH  Accessed on 13^th^ November 2025: <https://evek.biz/materials/chugun-chg8d3.html> |
| Gray Cast Iron | 2.5-4 | 1-3 | 0.1-1.2 | - | - | - | - | - | - | BCI Solutions, Inc.  Accessed on 13^th^ November 2025: <https://www.bcisolutions.com/gray-cast-iron#:~:text=Gray%20Cast%20Iron%2C%20also%20known,the%20iron%20it's%20gray%20color>. |

S-Table 16 Carbon and sulfur content (in wt%) of slag product from 2kg smelting

| **Elements** | **Before Roasting** | **After Roasting** |
| --- | --- | --- |
| C | 14.16 | 0.86 |
| S | 0.61 | 0.58 |
